# Enhanced binding of an HU homologue under increased DNA supercoiling preserves chromosome organisation and sustains *Streptomyces* hyphal growth

**DOI:** 10.1093/nar/gkac1093

**Published:** 2022-11-24

**Authors:** Agnieszka Strzałka, Agnieszka Kois-Ostrowska, Magda Kędra, Tomasz Łebkowski, Grażyna Bieniarz, Marcin J Szafran, Dagmara Jakimowicz

**Affiliations:** Molecular Microbiology Department, Faculty of Biotechnology, University of Wroclaw, Wroclaw, Poland; Molecular Microbiology Department, Faculty of Biotechnology, University of Wroclaw, Wroclaw, Poland; Molecular Microbiology Department, Faculty of Biotechnology, University of Wroclaw, Wroclaw, Poland; Molecular Microbiology Department, Faculty of Biotechnology, University of Wroclaw, Wroclaw, Poland; Molecular Microbiology Department, Faculty of Biotechnology, University of Wroclaw, Wroclaw, Poland; Molecular Microbiology Department, Faculty of Biotechnology, University of Wroclaw, Wroclaw, Poland; Molecular Microbiology Department, Faculty of Biotechnology, University of Wroclaw, Wroclaw, Poland

## Abstract

Bacterial chromosome topology is controlled by topoisomerases and nucleoid-associated proteins (NAPs). While topoisomerases regulate DNA supercoiling, NAPs introduce bends or coat DNA upon its binding, affecting DNA loop formation. *Streptomyces*, hyphal, multigenomic bacteria known for producing numerous clinically important compounds, use the highly processive topoisomerase I (TopA) to remove excessive negative DNA supercoils. Elongated vegetative *Streptomyces* cells contain multiple copies of their linear chromosome, which remain relaxed and relatively evenly distributed. Here, we explored how TopA cooperates with HupA, an HU homologue that is the most abundant *Streptomyces* NAP. We verified that HupA has an increased affinity for supercoiled DNA *in vivo* and *in vitro*. Analysis of mutant strains demonstrated that HupA elimination is detrimental under high DNA supercoiling conditions. The absence of HupA, combined with decreased TopA levels, disrupted chromosome distribution in hyphal cells, eventually inhibiting hyphal growth. We concluded that increased HupA binding to DNA under elevated chromosome supercoiling conditions is critical for the preservation of chromosome organisation.

## Graphical Abstract

**Graphical Abstract:**
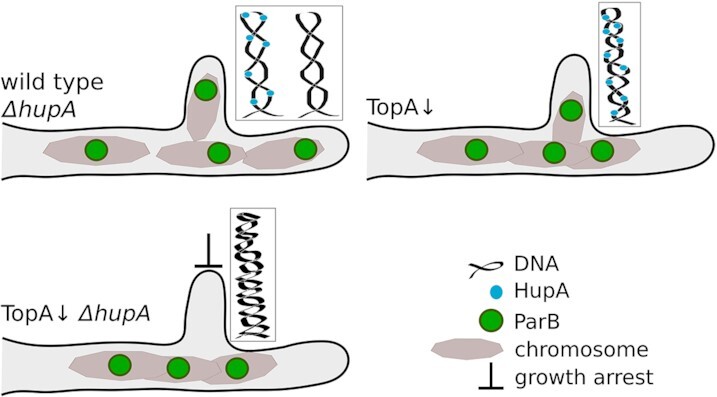
TopA and HupA cooperate in maintenance of chromosomal distribution in *Streptomyces coelicolor* vegetative hyphae.

## INTRODUCTION

Bacterial chromosome architecture is strictly controlled at all time points in the cell cycle. Chromosomal DNA is organised as independently supercoiled plectonemic loops (microdomains) that are assembled into larger domains (chromosome interaction domains) and eventually into macrodomains. In *Escherichia coli*, the size of the Ori, Ter, Left and Right macrodomains exceeds 1 Mb (1), while the average length of the DNA fragment in the microdomain is estimated to be ∼10 kb (2). The domain boundaries are determined by transcriptional activity and DNA-binding proteins. Two types of proteins that contribute to domain organisation are nucleoid-associated proteins (NAPs) and topoisomerases. Topoisomerases control supercoiling by removing excessive positive (type II topoisomerases, e.g. gyrases) or negative (type I topoisomerases, e.g. TopoI/TopA) supercoils that are generated by transcription or replication. On the other hand, small, positively charged NAPs coat or bridge DNA strands, inducing bends or stabilising DNA loops. The most abundant and conserved NAPs in bacterial cells are HU homologues (3). HU homologues form dimers and bind DNA without sequence specificity but may exhibit a preference for either AT-rich or supercoiled, single-stranded DNA or nicked regions and four-way junctions (4–9). Interestingly, limited evidence shows that HU homologues can influence topoisomerase activity. In *Mycobacterium smegmatis*, HupB was shown to alter TopA activity *in vitro* in a concentration-dependent manner (10). HU homologues from *E. coli*, *Thermotoga maritima* and *Streptococcus pneumoniae* have also been shown to increase negative supercoiling of previously relaxed DNA molecules (6,9,11). Moreover, the *E. coli* relaxation-deficient topoisomerase mutant *topA10* could not be combined with a deficiency of HU, while an overproduction of HU resulted in increased supercoiling of the nucleoid (12). These observations suggest an interplay between HU and topoisomerases.

*Streptomyces* are soil-dwelling bacteria valued as producers of antibiotics. They undergo a complex life cycle that encompasses mycelial vegetative growth and sporulation. During vegetative growth, *Streptomyces* hyphal cells extend apically and branch through the emergence of new tips at the lateral cell wall, developing a dense hyphal network. Replication of the *Streptomyces* linear chromosome is not directly followed by cell division, and consequently, elongated vegetative hyphal cells contain multiple chromosomal copies that remain uncondensed and are distributed along the cell (13). Efficient extension of vegetative hyphae requires anchoring of the apical chromosome at the tip, which is established by segregation proteins ParA and ParB. ParB binds in the proximity of the *oriC* region of each chromosome and forms large nucleoprotein complexes called segrosomes. When the branch emerges, one of the ParB complexes becomes anchored at the hyphal tip due to its interaction with tip-localised ParA (13). During sporulation, multiple chromosomes are uniformly aligned along sporogenic hyphae (14). At this stage, the ParA and ParB proteins are responsible for their distribution but also for DNA compaction through the recruitment of SMCs (15). Markedly, segrosomes have also been found to recruit TopA, which is presumably required to relieve topological tension generated by the formation of ParB complexes and assists in their separation (16). Interestingly, *Streptomyces* TopA is their sole and essential highly processive DNA relaxase (17). Its decreased level increases negative DNA supercoiling, severely affecting growth and inhibiting sporulation (16,18). Increased DNA supercoiling slows vegetative growth and affects chromosome distribution in vegetative hyphae (16,19). Moreover, TopA depletion resulted in transcriptional changes in ∼7% of *S. coelicolor* genes, including genes encoding two HU homologues (20).

HupA, a close homologue of *E. coli* HU, is the most abundant *S. coelicolor* NAP (20,21). HupS, the second *Streptomyces* HU homologue with a C-terminal extension, is much less abundant than HupA throughout vegetative growth, but its levels increase during sporulation (22). The cooperation between these two proteins or their heterodimerization has not yet been described. The elimination of any HU homologue only moderately affects *Streptomyces* growth. HupA deletion was shown to reduce the growth rate in *S. lividans*, while HupS deletion in *S. coelicolor* and *S. venezuelae* disrupted chromosome organisation during sporulation, leading to the formation of elongated spores that were sensitive to temperature stress (15,22,23). While HupS binding has been shown to be increased at terminal regions of the *S. venezuelae* linear chromosome (15), little is known about HupA binding and its dependence on DNA supercoiling.

Given the *Streptomyces* requirement to distribute multiple chromosomes in hyphal cells to ensure hyphal extension and branching, we expected that the chromosomal topology may be critical for hyphal growth. Based on HupA abundance and shared properties of the HU homologues, we hypothesized that HupA may be engaged with TopA to maintain proper DNA topology during *Streptomyces* hyphal growth. Thus, in this work, we sought to examine the role of HupA in *S. coelicolor* chromosome organisation and to determine whether it cooperates with TopA. To this end, we confirmed that increased DNA supercoiling leads to increased binding of HupA to DNA. Additionally, we depleted TopA in a *hupA* deletion background, analysed the growth of the mutant strain and examined the effects of mutations on chromosome distribution in hyphal cells. We confirmed that the presence of HupA is critical for maintaining chromosome distribution in hyphae under elevated supercoiling conditions. Taken together, our results suggest that HupA aids in preserving chromosome structure.

## MATERIALS AND METHODS

### Growth conditions and genetic modifications of bacterial strains

The *E. coli* and *S. coelicolor* strains used are listed in Supplementary Table S1. The culture conditions, antibiotic concentrations, and transformation and conjugation methods followed the general procedures for *E. coli* (24) and *Streptomyces* (25). For plate cultures of *S. coelicolor* strains, minimal medium supplemented with 1% mannitol (MM) or soy flour medium (SFM) was used. For the growth curves, *S. coelicolor* strains were cultured in microplates in 300 μl of YEME/TSB or ‘79’ medium (26), inoculated with spores to reach 0.01 U/ml (1 U of spores increases medium absorbance by 1) for 72 h at 30°C using a Bioscreen C (Automated Growth Curves Analysis System, Growth Curves USA), with five experimental replicates for each strain. The optical density (OD_600_) of the cultures was measured every 10 or 20 min.

The construction of the mutant *S. coelicolor* strains is described in the Supplementary Information. DNA manipulations were carried out using standard protocols. The genetic modifications of the obtained strains were verified by PCR and sequencing. The oligonucleotides used for PCR and the vector constructions are listed in Supplementary Table S2. Isolation and visualization of the supercoiled plasmid pWHM3Hyg was performed according to the protocol described in (27).

### HupA purification and electrophoretic mobility shift assay

The expression vector for HupA protein production was created by cloning the *hupA* gene, which was amplified using primers HupASco_fw and HupASco_rv, into a pGEX-6P-2 vector using the BamHI and XhoI restriction enzymes. The obtained expression plasmid was transformed into the *E. coli* BL21 strain. For protein overproduction, the cells were grown to OD600 ∼0.4 at 37°C. Then, isopropyl-β-d-thiogalactopyranoside (IPTG) was added to a final concentration of 0.3 mM, and the culture was continued for 4 h at 37°C. The cells were collected by centrifugation and resuspended in HA buffer (50 mM Tris–HCl, pH 8.0, 100 mM NaCl, 1 mM DTT and 10% glycerol). A fast protein liquid chromatography (FPLC) system with HP columns (GE Healthcare) was used to purify the recombinant protein from the cell lysate. Cells from 1 litre of culture were lysed by sonication in 50 ml of HA buffer, and the cellular lysate was clarified by centrifugation (25 000 g, 45 min, 4°C). The obtained lysate was incubated (4°C, 16 h) with a 0.5 ml bed volume of affinity resin. The resin was washed twice with 10 ml HA buffer and once with 10 ml PreS buffer (50 mM Tris–HCl, pH 7.5, 150 mM NaCl, 1 mM DTT, 1 mM EDTA). Resin-bound proteins were subjected to PreScission protease treatment overnight, according to the manufacturer's protocol (GE Healthcare). The resin was centrifuged (500 g, 5 min, 4°C), and the supernatant (containing proteins of interest) was collected in several fractions, followed by desalting using a Zeba Spin Desalting Column (Thermo Scientific) equilibrated with buffer B (50 mM NaH_2_PO_4_, pH 8.1, 300 mM NaCl and 10% glycerol). The purified protein was used in the EMSA experiment. Various HupA concentrations (0–40 μM) were incubated with 120 ng (3.6 nM) of pUC19 plasmid in buffer B for 3 min at room temperature, followed by incubation for 15 min at 37°C. The reaction mixture was then resolved in 1% agarose in Tris-acetate-EDTA (TAE) buffer for 14–16 h at a low voltage (2 V/cm). The plasmid DNA was visualized by staining the gel in ethidium bromide/TAE solution at room temperature for 30 min. Fluorescence signals were detected using a ChemiDoc instrument (Bio-Rad). For binding to relaxed DNA, the pUC19 plasmid, isolated using the Plasmid Midi AX kit (A&A Biotechnology), was previously relaxed by *E. coli* topoisomerase I (New England BioLabs) according to the manufacturer's instructions, followed by topoisomerase I inactivation at 65°C.

### DNA relaxation assay

The DNA relaxation reaction was carried out as previously described (17) with minor changes. Briefly, a mixture (16 μl) containing 200 ng (6 nM) of pUC19 plasmid DNA was incubated with the indicated amounts (corresponding to 30, 60 and 120 nM protein concentrations) of *S. venezuelae* TopA enzyme (92% identity with *S. coelicolor* TopA) for 15 min at 37°C. Next, increasing amounts (corresponding to 1.0, 2.0, 4.0 and 8.0 μM protein concentrations) of *S. coelicolor* HupA protein were added to the reaction mixtures as indicated, and the DNA binding reaction was carried out at 20°C for 15 min. Alternatively, both TopA and HupA were added simultaneously to the reaction mixture, followed by incubation for 15 min at 37°C. The reaction products, without deproteinization, were subjected to 1% agarose electrophoresis in 1× TBE buffer at 20°C for 16 h at a low voltage (2–3 V/cm), and the gel was stained with ethidium bromide. Fluorescence signals were detected using a ChemiDoc instrument (Bio-Rad).

### Chromatin immunoprecipitation (ChIP-seq) and bioinformatics analysis

For chromatin immunoprecipitation, *S. coelicolor* cultures (three independent biological repeats for each analysed strain) were grown in 50 ml liquid YEME/TSB medium at 30°C with shaking at 180 rpm. After 18 h of growth, the cultures were crosslinked with 1% formaldehyde for 30 min and blocked with 125 mM glycine. After washing with PBS (twice), the pellet was resuspended in 750 μl of lysis buffer (10 mM Tris–HCl, pH 8.0, 50 mM NaCl, 14 mg/ml lysozyme, protease inhibitor (Pierce)) and incubated at 37°C for 1 h. Next, the samples were sonicated to shear the DNA, and the length of the obtained fragments was verified by gel electrophoresis to be 300–500 bp. After centrifugation, 25 μl of each sample was stored to be used later as ‘input’ samples. The remaining supernatant was mixed with magnetic beads (60 μl) coated with an anti-FLAG M2 antibody (Merck). Immunoprecipitation was carried out overnight at 4°C. Next, the magnetic beads were washed twice with IP buffer (50 mM Tris–HCl pH 8.0, 250 mM NaCl, 0.8% Triton X-100, protease inhibitors (Pierce)) and once with TE buffer (Tris–HCl, pH 7.6, 10 mM EDTA 10 mM). The DNA was released by overnight incubation of the beads resuspended in 150 μl IP elution buffer (50 mM Tris–HCl pH 7.6, 10 mM EDTA, 1% SDS, also added to the ‘input’ samples) at 65 °C. After centrifugation, proteinase K (Roche, 100 μg/ml) was added to the supernatants, and the samples were incubated for 90 min at 55 °C. DNA was extracted with phenol and chloroform and subsequently precipitated with ethanol overnight. The precipitated DNA was dissolved in nuclease-free water (10 μl). The DNA concentration was quantified using a Qubit 1X dsDNA HS Assay kit (Thermo Fisher).

DNA sequencing was performed by Fasteris SA (Switzerland) using the Illumina ChIP-Seq TruSeq protocol, which included quality control, library preparation and sequencing from both ends (2  ×  150 bp) of the amplified fragments. Sequencing data for the HupA-FLAG ChIP-Seq experiment were collected and preprocessed by Fasteris SA using HiSeq Control Software 3.4.0.38, RTA 2.7.7, and bcl2fastq2.17 v2.17.1.14 software. The ChIP-seq reads were mapped using the Qiagen CLC – Genomics Workbench 8.5.1. The total number of mapped reads was >10^7^/library on average. The differentially bound regions were found using the R packages *csaw* and *edgeR* (28–30) as described (31) or using the MACS3 program (32). Briefly, when using *edgeR*, the reads were counted using a 69-bp-long window with a 23 bp slide. The peaks were filtered using the local method, which compares the number of reads in the region to the background computed in the surrounding window of 2000 bp. Only peaks with a log_2_-fold change >2 were retained for further analyses. A quasi-likelihood negative binomial generalized log-linear model was fitted to the count data. Regions that were <100 bp apart were merged, and the combined p value for each region was calculated. Only regions with FDR values below the 0.05 threshold were considered differentially bound between two samples. For MACS3 analysis, peaks were detected using input files as controls with broad and nomodel settings. Only peaks with scores (calculated as –log_10_ of *q*-value) higher than 200 were retained for further analysis.

### RNA isolation, reverse transcription and quantitative PCR (RT−qPCR)

RNA was isolated from *S. coelicolor* cultures cultivated in 5 ml YEME/TSB liquid medium for 24 h. Mycelia were collected by centrifugation, frozen and stored at -70°C for subsequent RNA isolation. RNA was isolated using the RNeasy Mini Kit (Qiagen) following the manufacturer's instructions, digested with TURBO DNase I (Invitrogen) and checked for chromosomal DNA contamination using PCR. A total of 500 ng of RNA was used for cDNA synthesis using the Maxima First Strand cDNA synthesis kit (Thermo Fisher Scientific) in a final volume of 20 μl. The obtained cDNA was diluted to 100 μl and directly used for quantitative PCRs performed with PowerUp SYBR Green Master Mix (Applied Biosystems). The relative level of *hupA* was quantified using the comparative ΔΔCt method, and the *hrdB* gene was used as the endogenous control (StepOne Plus real-time PCR system, Applied Biosystems).

### Microscopy analysis

For time-lapse imaging, 10^–1^–10^–3^ spore dilutions were spotted onto cellophane membranes on MM solid medium supplemented with 1% mannitol and cultured for 2 h (for spore germination analysis) or 24 h (for vegetative growth analysis) before the start of the experiments. Image acquisition and data analysis were performed as previously described (13). Briefly, all experiments were carried out using a μ-dish (35 mm, Ibidi) covered with a block of agar and a Delta Vision Elite inverted microscope at 30°C with a 100 × 1.46 NA oil immersion objective, ultimate focus and Olympus IX71 camera. Images were acquired every 10 min using differential interference contrast (DIC) and EGFP (λ_ex/em_ 475/525 nm) and mCherry (λ_ex/em_ 575/625 nm) filters. The positions of the fluorescent complexes were identified using custom protocols involving Fiji software and the R package Peaks (Morhac M (2012). Peaks. R package version 0.2) (33) (code available at https://github.com/astrzalka/findpeaks). The germination frequency was determined by manually counting all spores visible in the field of view during microscopy experiments. The spores were assigned as nongerminating if no hyphae appeared during the entire 16-hour experiment, and hyphal growth was assigned as inhibited if, after initial hyphal extension, no hyphal extension was visible for more than 1 h.

For structured illumination microscopy (SIM), *S. coelicolor* spores were cultured on microscopy coverslips inserted at a 45° angle in MM solid medium containing 1% mannitol for 24 h and then fixed with a 2.8% paraformaldehyde/0.00875% glutaraldehyde mixture for 10 min at room temperature. The hyphae were imaged using a Zeiss Elyra 7 microscope equipped with an alpha Plan-Apochromat 100x/1.46 Oil DIC M27 objective. Images were acquired using a Z-stack (0.1 μm) and processed with ZEN and Fiji software. For DNA staining, after fixation, as described above, the samples were digested with lysozyme (2 mg/ml in 20 mM Tris–HCl supplemented with 10 mM EDTA and 0.9% glucose) for 2 min, washed with PBS, blocked with 2% BSA in a PBS buffer for 10 min and incubated with 0.1–1 μg/ml DAPI (4',6-diamidino-2-phenylindole) (Molecular Probes) for 60 min. Fluorescence microscopy was performed using a Zeiss Axio Imager Z1 fluorescence microscope equipped with a 100× oil immersion objective and DAPI Zeiss filter Set 49 (λ ex/em 365/445 nm).

Single-molecule tracking using PALM was performed with a Nanoimager-S fluorescence microscope equipped with a 1.4 NA 100× oil immersion objective. For imaging, *S. coelicolor* cultures were inoculated on coverslips inserted into MM agar supplemented with 1% mannitol at a 45° angle. After 24 h of incubation, the coverslips were washed with PBS and placed on low-fluorescence agarose (Bio-Rad) cubes. HupA-PAmCherry activation was controlled with a 405 nm laser (2–8% laser power) and excitation at 561 nm (15–20% laser power) with an exposure time of 30 ms. For each position, 20 000 frames were collected. Single-particle tracking was performed using Fiji and TrackMate software (34). The positions were linked if they appeared in consecutive frames within a 0.6 μm radius, and 1 missing frame was allowed for transient disappearance due to blinking or missed localization. To determine the molecular mobility, SMTracker software was used (35), which employs a Gaussian mixture model (GMM) to calculate the diffusion constants of proteins in multiple states, such as those bound to DNA versus those freely diffusing in the solution. Based on these results, a proportion of the constrained tracks was calculated for the 90 ms time frame.

## RESULTS

### HupA preferentially binds to negatively supercoiled DNA

**Figure 1.**
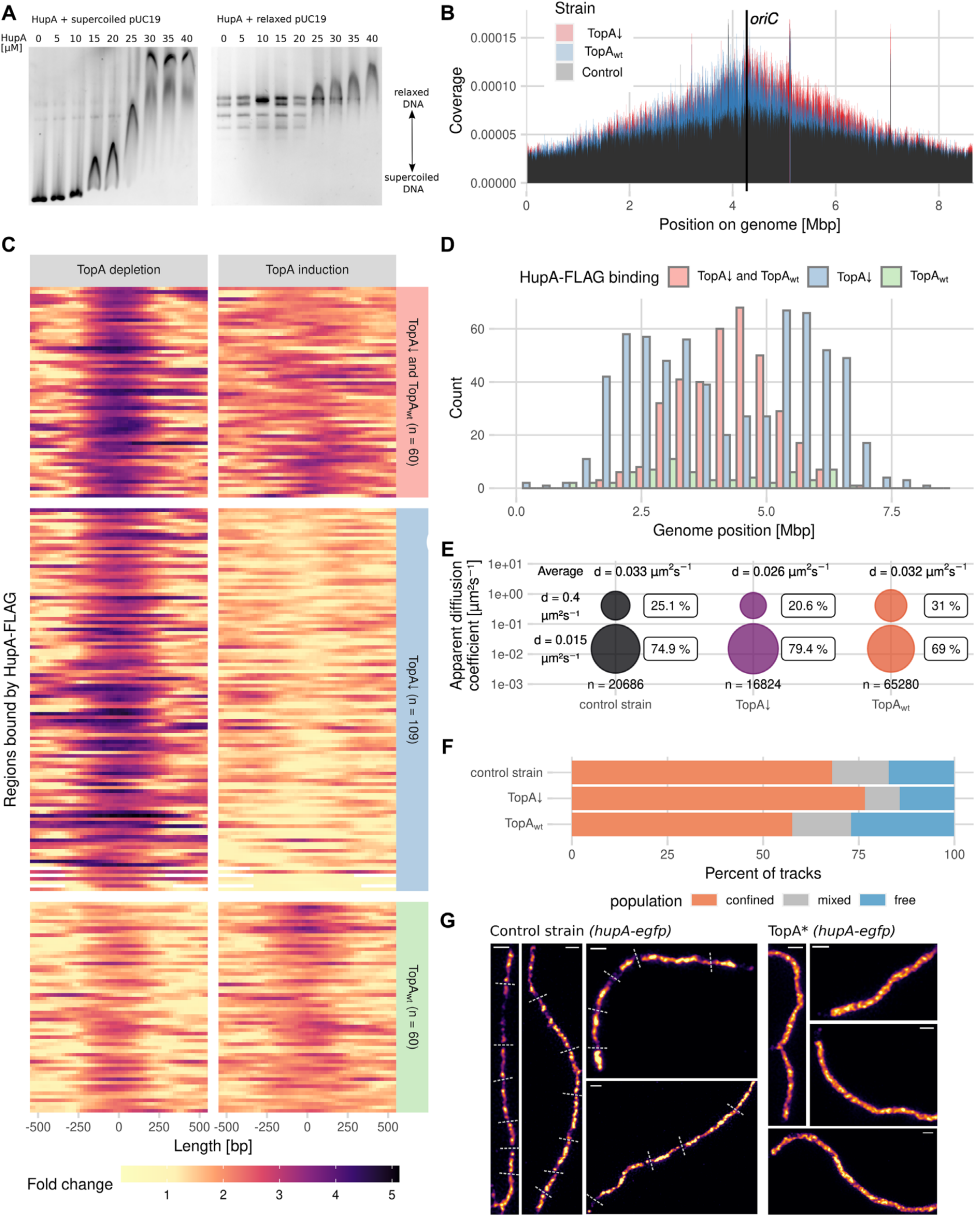
Dependence of *S. coelicolor* HupA–DNA binding on the DNA supercoiling level. (**A**) EMSA analysis of HupA (0–40 μM) binding to 3.6 nM pUC19 plasmid: supercoiled (left panel) and relaxed by *E. coli* topoisomerase I (right panel). The positions of the supercoiled and relaxed topoisomers are indicated. (**B**) ChIP-seq-detected binding of HupA-FLAG to the *S. coelicolor* chromosome in the TopA-depleted strain (TopA*, ASMK032, red) and the same strain with restored TopA levels (2 μg/ml thiostrepton, blue) compared to the control strain that did not produce HupA-FLAG (TopA*, PS04, black). Reads were counted in 200 bp bins and divided by the total number of reads to obtain the chromosomal coverage. The results from three (ASMK032) and two (PS04) biological repeats were averaged. (**C**) Heatmaps of 229 regions differentially bound by HupA-FLAG in the TopA-depleted strain (TopA*, ASMK032) (left panel) and the same strain with restored TopA levels (right panel) (2 μg/ml thiostrepton). Colours represent a fold change against the control strain with restored TopA levels (PS04 grown in the presence of 2 μg/ml thiostrepton). The number of reads was fitted by the glmQL model from the *edgeR* package. (**D**) Chromosomal positions of the regions differentially bound by the HupA-FLAG protein in the TopA-depleted HupA-FLAG strain (TopA*, ASMK032, blue) compared to the same strain with restored TopA levels (green) and regions bound by HupA-FLAG in both conditions (red) identified by the MACS3 program. (**E**) Mobility of HupA-PAmCherry molecules indicated by PALM tracking in the control strain (ASMK015, black) and the TopA-depleted strain (TopA*, ASMK035) grown without the *topA* inducer (purple) and with the *topA* inducer (red). The bubble plots show the fit to a two-component model by a Gaussian mixture model from the SMTracker software. The HupA-PAmCherry molecules were divided into an immobile fraction (bottom circles) and mobile fraction (top circles) based on their apparent diffusion coefficients. The total number of analysed tracks and average apparent diffusion coefficient are shown on the plot for each strain. (**F**) Percentage of HupA-PAmCherry PALM tracks that were classified as confined (orange), free (blue) or mixed (light grey) by SMTracker software based upon the fit of the Gaussian mixture model at three consecutive timepoints (90 ms) in the control strain (ASMK015), the TopA-depleted strain (TopA*, ASMK035) and the TopA-depleted strain with restored TopA levels (2 μg/ml thiostrepton). (**G**) SIM images of HupA-EGFP fluorescence in the control (K306) and TopA-depleted strains (TopA*, AS40) in 24-h vegetative hyphae. Dashed lines shown on the control image indicate possible separated chromosomal copies. Scale bar, 1 μm.

First, we examined whether *S. coelicolor* HupA exhibits a preference for supercoiled or relaxed DNA. We used an electrophoretic mobility shift assay (EMSA) to test the binding of recombinant HupA (digested from a GST fusion, Figure S1A) to the negatively supercoiled plasmid pUC19 and the same plasmid relaxed by *E. coli* TopA. While the supercoiled plasmid was bound by HupA at a 15 μM concentration, the relaxed plasmid was not bound at this protein concentration, and a 40 μM HupA concentration was required to shift all relaxed plasmid topoisomers (Figure 1A). This finding confirms that *S. coelicolor* HupA prefers to bind to negatively supercoiled DNA rather than relaxed DNA. Additionally, to investigate the potential cooperation between HupA and TopA, we tested whether HupA affects TopA activity or DNA supercoiling levels *in vitro*. This experiment was performed at a lower NaCl concentration than used in the EMSA experiment (150 mM versus 300 mM in the EMSA experiment) since previous studies have shown that *E. coli* HU increased DNA supercoiling at physiological concentrations of NaCl (150 mM) (36). Moreover, in buffer containing 300 mM NaCl, we did not observe any changes in DNA supercoiling caused by HupA addition (Figure 1A, right panel). The supercoiled DNA was either relaxed first by TopA, and then HupA was added to the reaction mixtures to concentrations ranging from 1 to 4 μM (Figure S1B), or both proteins were added simultaneously (Figure S1C). We established that the presence of HupA in the TopA reaction mixture resulted in an increased content of highly supercoiled plasmid topoisomers (Figure S1 B, C), which may be due to inhibited TopA activity or reverted DNA relaxation. Interestingly, the concentration of HupA required to increase DNA supercoiling in the presence of TopA was lower (2 μM) than that required to shift DNA in the EMSA experiment, although this difference could be due to the different buffer compositions used in the EMSA and topoisomerase assays. Nevertheless, these experiments showed that HupA not only bound preferably to highly supercoiled DNA but could also affect TopA activity and/or DNA topology.

Since *S. coelicolor* HupA showed a preference for negatively supercoiled DNA *in vitro*, we hypothesized that its binding to DNA would be enhanced in the TopA-depleted strain due to increased chromosome negative supercoiling. To verify this hypothesis, we set out to perform ChIP-seq analysis of HupA-FLAG at different chromosome supercoiling levels. Placing the *topA* gene under the control of a thiostrepton-inducible promoter allowed TopA depletion in the absence of the inducer, with levels reduced to ∼20 times lower than in the wild type (16). In cultures grown in the presence of thiostrepton at a 2 μg/ml concentration, TopA levels and chromosome supercoiling were restored to approximately wild-type levels, while a lower concentration (0.2 μg/ml) was sufficient for restored strain growth in liquid medium (16,19,27). Analysis of the strains expressing *hupA-FLAG* as a single *hupA* gene copy in the wild-type background (*ΔhupA hupA-FLAG*, ASMK012) and in the strain with a controlled TopA level (TopA* *ΔhupA hupA-FLAG*, ASMK034) suggested that HupA-FLAG fully complements *hupA* deletion at the normal chromosome supercoiling level, but complementation was only partial under increased supercoiling (Figure S2A, B, E), indicating that FLAG could somewhat affect HupA function. Markedly, transcript analysis indicated a lowered level of *hupA-FLAG* transcript under normal supercoiling compared to *hupA* transcript (Figure S3D). Moreover, western blot analysis of HupA-FLAG showed that its level was significantly lower in the TopA depletion strain than in the same strain with restored TopA levels (TopA* *ΔhupA hupA-FLAG*) or the control strain (*ΔhupA hupA-FLAG*) (Figure S3C). This lowered level of HupA-FLAG may be explained by altered transcriptional regulation of the modified gene, possibly by autoregulation and/or supercoiling. Lowering the amount of HupA-FLAG, possibly coupled with lowering the affinity of this protein to DNA, resulted in no detectable HupA-FLAG binding using the ChIP-seq method. Therefore, we used a strain with *hupA-FLAG* as an additional copy of the *hupA* gene (TopA* *hupA-FLAG*, ASMK032). An additional copy of the *hupA-FLAG* gene did not affect *S. coelicolor* growth in a liquid medium (Figure S2C). Western blot analysis showed that the level of the HupA–FLAG protein in this strain was somewhat higher than or similar to that in the strain with *hupA-FLAG* as only the *hupA* gene (TopA* *ΔhupA hupA-FLAG* or *ΔhupA hupA-FLAG*). Markedly, in the strain with *hupA-FLAG* as an additional *hupA* copy, the level of HupA-FLAG decreased upon TopA depletion, similar to the strain with a single *hupA-FLAG* copy (Figure S3C). Thus, we used strain *hupA-FLAG* as an additional *hupA* copy (TopA* *hupA-FLAG*, ASMK032) with depleted TopA levels to examine HupA-FLAG binding. As a control, the same strain was cultured with the addition of a *topA* gene inducer (2 μg/ml thiostrepton) to maintain approximately wild-type TopA levels. Additionally, the strain with unmodified *hupA* and restored TopA levels (TopA*, PS04, cultured in the presence of 2 μg/ml thiostrepton) was used as the negative control. ChIP-seq analysis showed the binding of HupA-FLAG (possibly enhanced by the presence of untagged HupA) to DNA. However, none of the identified sites appeared to be considerably more enriched than the neighbouring regions, indicating that HupA binds nonspecifically to DNA (Figure 1B). This ChIP-seq dataset was analysed using two programs: the R package edgeR, which detects only peaks with a high fold change against the control strain, and MACS3, which should also locate sites weakly bound by HupA-FLAG.

An analysis of HupA-FLAG binding to DNA revealed that more HupA-FLAG-bound regions were present around *oriC* (between 3 and 6 Mbp) than in the negative control strain (Figure 1B). Under elevated chromosome supercoiling in the TopA-depleted strain, HupA-FLAG showed more binding than in the strain with restored TopA levels (Figure 1B, C). While the sites that were bound independently of supercoiling were located around *oriC*, the regions whose binding by HupA-FLAG was dependent on chromosome supercoiling level were distributed further away from the chromosome centre but not in the arm regions (Figure 1D). An analysis of the ChIP-seq data using Bioconductor edgeR package revealed the presence of 229 regions bound by HupA-FLAG, with 109 regions bound only under increased DNA supercoiling, 60 regions bound by HupA-FLAG in the strain with the TopA level restored, and 60 regions bound independently of supercoiling conditions (Figure 1C). An analysis of HupA-FLAG binding using the MACS3 program delivered an additional 1076 probable binding sites, confirming half of the previously identified regions. Among the sites identified by MACS3 analysis, 362 were bound independently of the TopA level, 649 were bound under TopA depletion conditions, and only 65 were bound exclusively when the TopA level was restored. Given the published evidence that HU homologues show a preference for AT-rich regions (37), we verified the identified regions by calculating the percentage of AT base pairs. Indeed, we found an increase in the median AT% for both edgeR and MACS3 detected sites (Figure S4). This finding suggests an overall increase in HupA binding under elevated chromosome supercoiling, even though the levels of HupA did not increase.

To further analyse the binding of individual HupA molecules to DNA *in vivo* and to exclude the potential modified binding pattern resulting from the presence of HupA-FLAG as the additional HupA copy, we used a complementary approach, i.e. photoactivation localization microscopy (PALM). In this experiment, we tracked the mobility of the fusion protein HupA-PAmCherry in the wild-type background and in a strain with controlled TopA levels (in this experiment, both strains possessed only a single copy of the *hupA* gene: *ΔhupA hupA-PAmCherry*, ASMK015 and TopA* *ΔhupA hupA-PAmCherry*, ASMK035, respectively). Both strains that possessed only *hupA-PAmCherry* genes grew similarly to the control strains that encoded the native *hupA* gene; however, the *hupA-PAmCherry* transcript level measured by RT−qPCR was lower than the *hupA* transcript level in the control strains (Figures S2A, C and S3D), similar to that observed for *hupA-FLAG*.

The calculation of the apparent diffusion coefficients of the HupA-PAmCherry molecules showed that TopA depletion increased the percentage of immobile HupA-PAmCherry molecules (*d* = 0.015 ± 0.000015 μm^2^ s^–1^ for the immobile fraction and *d* = 0.400 ± 0.0013 μm^2^ s^−^^1^ for the mobile fraction) to 79.4% from 74.9% in the wild-type background. Increasing the TopA level (2 μg/ml thiostrepton) had the opposite effect, decreasing the fraction of immobile HupA-PAmCherry molecules to 69% (Figure 1E). The calculated average diffusion coefficient of the HupA-PAmCherry molecules was also lower in the TopA-depleted strain (*d* = 0.026 μm^2^ s^−1^) than in the wild-type or TopA-induced strains (*d* = 0.033 μm^2^ s^−1^ and *d* = 0.032 μm^2^ s^−1^, respectively). Additionally, in the TopA-depleted strain, the fraction of HupA-PAmCherry molecules that remained stationary for three consecutive time points was higher (76.6%) than that in either the wild-type or TopA-induced strains (68% and 57.6%, respectively) (Figure 1F). This observation supports the more stable binding of HupA to DNA under elevated supercoiling conditions *in vivo*.

Finally, we analysed the HupA-EGFP localization pattern in wild-type (*hupA-egfp*, K306) and TopA-depleted strains (TopA* *hupA-egfp*, AS40), which both expressed *hupA-egfp* from the native locus. Structured illumination microscopy (SIM) observations of HupA-EGFP showed an altered HupA binding pattern in the mature hyphae of the TopA-depleted strain. While the boundaries of the HupA-EGFP signal could be distinguished in the control strain, in the TopA-depleted strain, the HupA-EGFP signal was more uniformly spread along the hyphae (Figure 1G). The observed change in the HupA-EGFP pattern could be due to differences in its binding under TopA depletion or differences in chromosome organisation and/or distribution under such conditions.

In summary, we demonstrated that *S. coelicolor* HupA preferentially binds to negatively supercoiled DNA *in vitro*. This result was corroborated by the ChIP-seq experiment results that showed increased binding of HupA-FLAG to DNA in the TopA-depleted strain and the PALM experiment results showing decreased mobility of HupA-PAmCherry molecules.

### HupA deletion delays growth and is detrimental when combined with TopA depletion

**Figure 2.**
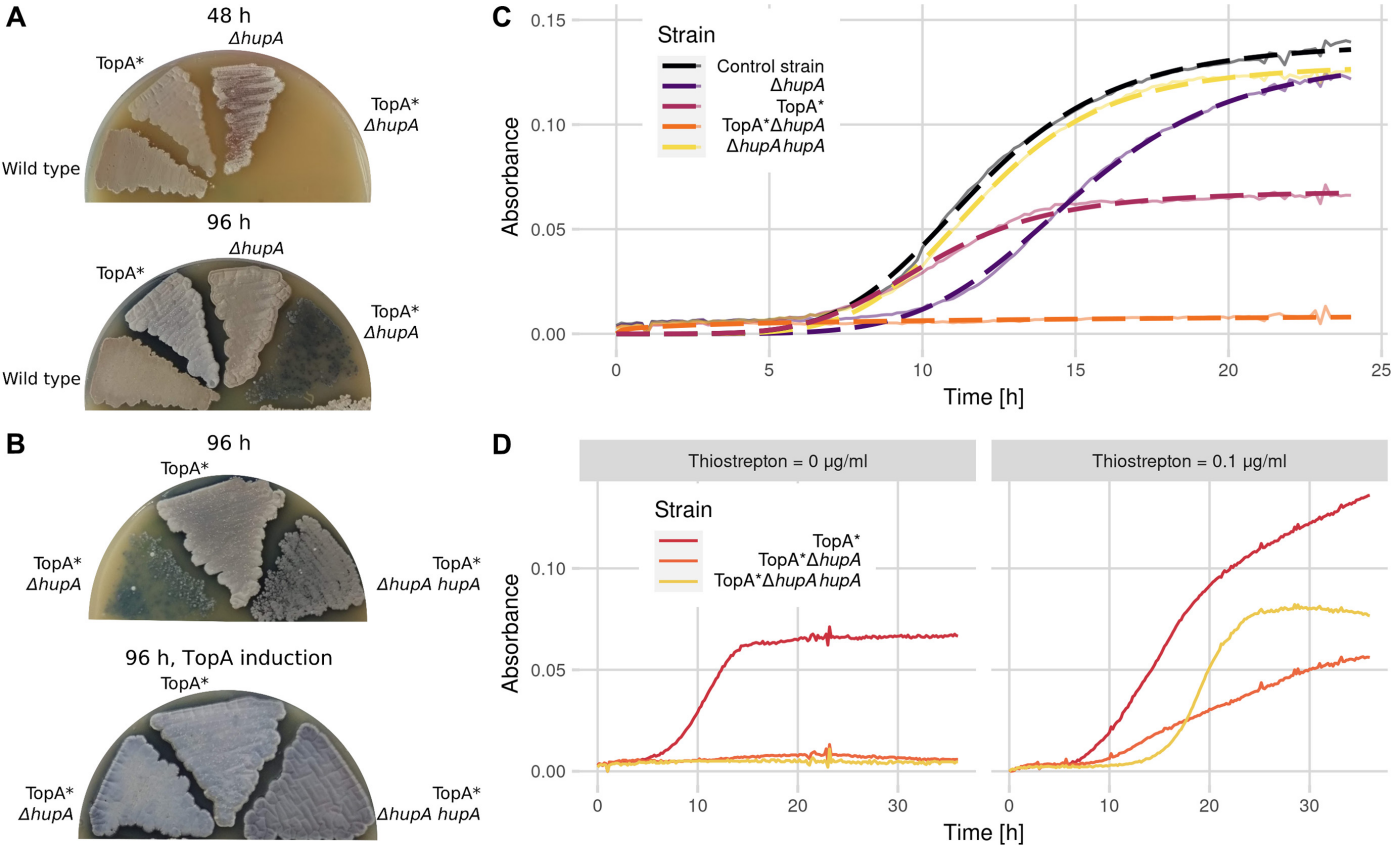
Phenotypic effects of *hupA* deletion in wild-type and TopA-depleted *S. coelicolor* strains. (**A**) Slower development of the Δ*hupA* (ASMK011) and TopA-depleted Δ*hupA* (TopA* Δ*hupA*, ASMK031) strains compared to the TopA-depleted (TopA*, PS04) and wild-type paternal strains (M145) at 48 and 96 h of growth on SFM medium. (**B**) Slower development of the TopA-depleted Δ*hupA* strain (TopA* Δ*hupA*, ASMK031) compared to the TopA-depleted strain (TopA*, PS04) and TopA-depleted Δ*hupA* strain complemented in trans with *hupA* (TopA* Δ*hupA hupA*, ASMK033) at 96 h of growth on SFM (upper panel) compared to growth of the same strains in the presence of the *topA* expression inducer (2 μg/ml thiostrepton) (lower panel). The grey pigment is characteristic of fully developed aerial hyphae, a white colony colour indicates an inhibition of *S. coelicolor* sporulation, and blue results from the production of actinorhodin. (**C**) Growth of the Δ*hupA* (ASMK011, purple), TopA-depleted (TopA*, PS04, pink), TopA-depleted Δ*hupA* (TopA* Δ*hupA*, ASMK031, orange), Δ*hupA* complemented in trans with *hupA* (ASMK013, yellow) and wild-type (M145, black) strains cultured in YEME/TSB medium. The semitransparent lines show the mean absorbance values obtained from five replicates, and the dashed lines correspond to the fit of the log-logistic growth model. (**D**) Growth of TopA-depleted (TopA*, PS04, pink), TopA-depleted Δ*hupA* strain (TopA* Δ*hupA*, ASMK031, orange), and complemented TopA-depleted Δ*hupA* deletion (TopA* Δ*hupA hupA*, ASMK033, yellow) strains cultured in YEME/TSB medium.

TopA depletion in *S. coelicolor* inhibits the growth of vegetative hyphae and blocks sporulation by impeding the maturation of white aerial hyphae into chains of grey spores (16) (Figure 2A, B). Additionally, increased chromosome supercoiling alters the expression of 7% of *S. coelicolor* genes, including *hupA*, whose transcription level increased 1.5-fold after TopA depletion (20). However, the analysis of the HupA-EGFP level in the strain with a controlled TopA level (EGFP insertion in the *hupA* native chromosomal locus, TopA* *hupA-egfp*, AS40 strain) showed that HupA-EGFP fluorescence remained stable despite changes in the TopA level (thiostrepton concentrations ranging from 0–1 μg/ml) at the same stage of growth as the earlier RNA-seq experiment (18 h, Figure S3 A). Interestingly, at 48 h, the level of HupA-EGFP decreased in the TopA-depleted strain compared to the control strain with wild-type supercoiling (Figure S3B, C). This experiment corroborated the above-described analysis of the HupA-FLAG level in the TopA-depleted strain but not earlier transcriptional analyses (20); the lack of transcriptional activation of the tagged *hupA* gene may be due to its modified regulation resulting from its genetic modification. However, our *in vitro* and *in vivo* experiments indicated that HupA binding was enhanced by increased DNA supercoiling. Since the function of HU homologues is associated with the stabilisation of plectonemic loops, we hypothesized that increased binding of HupA to negatively supercoiled DNA could compensate for decreased TopA levels. To test this hypothesis, we investigated how TopA depletion in a *hupA* deletion background affected the growth of *S. coelicolor*.

Deletion of *hupA* in the wild-type background (Δ*hupA*, ASMK011) only moderately inhibited *S. coelicolor* growth (Figure 2A, C). In plate cultures, in which *S. coelicolor* underwent a full differentiation cycle, *hupA* deletion delayed growth, while in liquid cultures, in which *S. coelicolor* grows only vegetatively, the Δ*hupA* mutant was retarded at an early stage of growth compared to the wild-type strain (ED50—time when culture reached 50% of maximal absorbance as measured by the log-logistic model: 15.1 ± 0.05 h for Δ*hupA* and 11.8 ± 0.05 h for the wild-type strain), while the relative slope of the growth curve was only moderately affected (−5.71 ± 0.14 and −5.08 ± 0.1, respectively) (Figure 2A, C). This growth curve analysis suggested that the influence of *hupA* deletion was more pronounced during spore germination and less extensive during vegetative growth. The growth was restored by complementation with the wild-type *hupA* gene delivered in plasmid pIJ170*hupA* (Figure 2C). RT-qPCR analysis of *hupA* expression also showed a similar level of transcription in both the control and complementation strains (Figure S3 D).

To investigate the effects of TopA depletion in the Δ*hupA* background, we deleted the *hupA* gene in a strain with controlled *topA* expression (TopA*, PS04). The TopA-depleted strain that lacked HupA (TopA* Δ*hupA*, ASMK031) exhibited severely inhibited growth in both liquid and plate cultures, and sporulation was not detectable even after prolonged incubation (Figures 2A, C, S5D). The introduction of the *hupA* gene using the integrative plasmid pIJ170*hupA* (TopA* Δ*hupA hupA*, ASMK033) partially complemented *hupA* deletion in the TopA-depleted strain, but only during growth in solid medium (Figure 2B, D). On the other hand, increasing TopA levels in the Δ*hupA* strain restored development to the same level as observed for the control strain (TopA*) and partially restored growth in liquid culture (Figure 2B, D). The lack of full complementation may result either from the presence/activity of additional protein(s) contributing to maintaining chromosome topology under particular growth conditions or modified levels of HupA or TopA. Interestingly, when compared to the wild-type strain, the growth of the Δ*hupA* strain was significantly delayed when cultured in the presence of the gyrase inhibitor novobiocin (Figure S2 D), indicating its increased sensitivity to any chromosome supercoiling imbalance. Given that *hupA* deletion resulted in diminished survival of the TopA-depleted strain, characterized by high DNA supercoiling, we aimed to test whether an elevated HupA level would enhance growth under such conditions. We placed *hupA* under a strong constitutive promoter (*ermhupA*, AS41), which increased the *hupA* transcript level twofold; however, the growth of the obtained strain in the presence of novobiocin remained similar to that of the wild-type strain (Figures S2E and S3D). This experiment, as well as difficulties with full complementation of the TopA-depleted Δ*hupA* strain, may suggest tight cooperation between TopA and HupA that requires their mutually adjusted levels.

Additionally, analysis of the supercoiling of the reporter plasmid pWHM3Hyg isolated from 48 h vegetative cultures of the TopA-depleted Δ*hupA* strain (TopA* Δ*hupA* pWHM3Hyg, ASMK036) and the TopA-depleted control strain (TopA*, pWHM3Hyg, MS11) showed a similar pattern of topoisomers in both strains (Figure S3E). This observation suggested that the average global DNA supercoiling level was not increased by *hupA* deletion in the TopA-depleted strain; however, it does not exclude the possibility that HupA locally preserves chromosomal organization under elevated supercoiling conditions, i.e. by protecting against supercoil spreading.

In summary, *hupA* deletion only moderately slowed *S. coelicolor* growth but led to severe growth inhibition when combined with TopA depletion.

### *hupA* deletion combined with TopA depletion impairs spore germination and disturbs chromosome distribution in hyphae

We hypothesized that the impaired growth of the TopA-depleted Δ*hupA* strain was due to a deeply altered nucleoid structure, which could affect transcription and/or impair the distribution of multiple nucleoids in *Streptomyces* hyphae. The slower growth of the mutant strains could be caused by either delayed spore germination or inhibited extension and branching of hyphae. To analyse the germination and growth of mutant strains at the single-cell level, we used time-lapse microscopy. The spores of the *hupA* deletion and reference strains with controlled TopA levels (TopA* Δ*hupA* and TopA*) were collected from cultures with restored TopA levels (2 μg/ml thiostrepton), and their germination and hyphal tube extension under TopA depletion was observed for 16 h.

**Figure 3.**
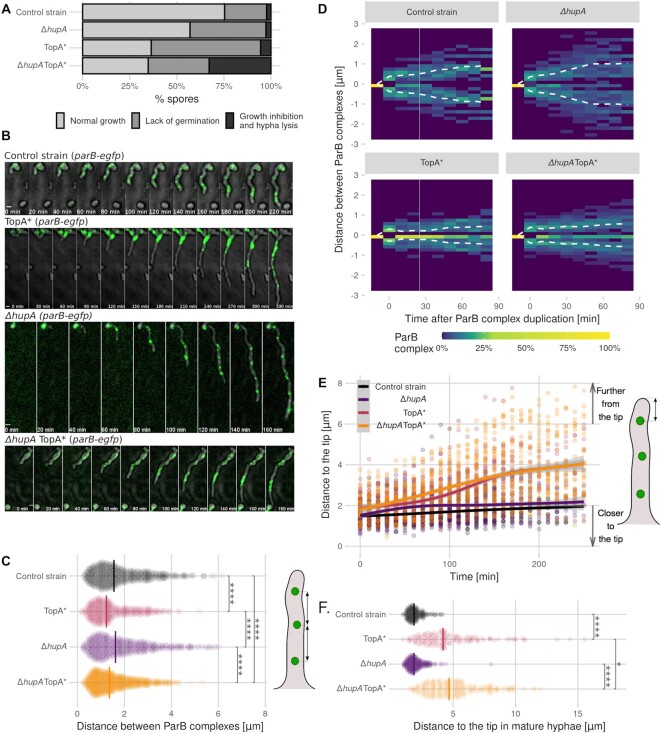
Influence of TopA depletion and *hupA* deletion on germination and chromosome distribution in early vegetative hyphae of *S. coelicolor*. (**A**) Percentage of spores that undergo normal germination, undergo growth inhibition upon germination (stop growing within 4 h of germination) or do not germinate during 16 h of observation under a time-lapse microscope for the control strain (AK101, 430 spores), TopA-depleted strain (TopA*, AS11, 434 spores), Δ*hupA* (ASMK02, 372 spores) and TopA-depleted Δ*hupA* (TopA* Δ*hupA*, ASMK05, 454 spores) (all in the *parB-egfp*, *dnaN-mCherry* genetic background) cultured on minimal medium. (**B**) Snapshots from the time-lapse analysis of the ParB complexes (green) in the germinating spores of the control strain (AK101), TopA-depleted strain (TopA*, AS11), *ΔhupA* strain (ASMK02) and TopA-depleted Δ*hupA* strain (TopA* Δ*hupA*, ASMK05) (all in the *parB-egfp* and *dnaN-mCherry* genetic background). The fluorescence images are merged with the DIC images (grey). Scale bar, 1 μm. (**C**) Distribution of distances between all ParB complexes in the germinating spores of the control strain (AK101, black, 35 hyphae), TopA-depleted strain (TopA*, AS11, pink, 32 hyphae), *ΔhupA* strain (ASMK02, purple, 32 hyphae) and TopA-depleted Δ*hupA* strain (TopA* Δ*hupA*, ASMK05, orange, 45 hyphae) (all in the *parB-egfp* and *dnaN-mCherry* genetic background). The distance measurements are shown as semitransparent points, with the vertical line indicating the median value. The schematic drawing of *S. coelicolor* hyphae on the right side indicates the measured distance. (**D**) Heatmaps showing the relative positions of the duplicated, tip-proximal ParB complexes in early vegetative hyphae for 90 min after duplication in 10-min intervals. The dashed line shows the average position of the ParB complexes in the control strain (AK101, 70 duplications), TopA-depleted strain (TopA*, AS11, 56 duplications), Δ*hupA* strain (ASMK02, 74 duplications) and TopA-depleted Δ*hupA* strain (TopA* Δ*hupA*, ASMK05, 116 duplications). (**E**) The change over time in the distance between the tip-proximal ParB complex and the tip of the hyphae in germinating spores of the control strain (AK101, black, 35 hyphae), TopA-depleted strain (TopA*, AS11, pink, 32 hyphae), Δ*hupA* strain (ASMK02, purple, 32 hyphae) and TopA-depleted Δ*hupA* strain (TopA* Δ*hupA*, ASMK05, orange, 45 hyphae) (all in the *parB-egfp* and *dnaN-mCherry* background). Lines show the model fitted with the loess algorithm. F. Distribution of the distances between the tip-proximal ParB complex and the hyphal tip in long hyphae (>10 μm) in the control strain (AK101, black, 150 hyphae), TopA-depleted strain (TopA*, AS11, pink, 132 hyphae), Δ*hupA* strain (ASMK02, purple, 204 hyphae) and TopA-depleted Δ*hupA* strain (TopA* Δ*hupA*, ASMK05, orange, 180 hyphae) (all in the *parB-egfp* and *dnaN-mCherry* background). A schematic drawing of the *S. coelicolor* hyphae shows the measured distance for Panels E and F. In Panels C and F, the statistical significance was assessed using the Wilcoxon test, with the following significance levels: *P ≤ 0.05, **P ≤ 0.01, ***P ≤ 0.001, ****P ≤ 0.0001.

Deletion of *hupA* severely inhibited spore germination; approximately 40% of spores failed to develop hyphal tubes within 16 h, while in the wild-type strain, 22% of spores did not germinate under the same experimental conditions. As previously observed (16), TopA depletion inhibited the germination of approximately 58% of spores. In the TopA-depleted Δ*hupA* mutant, approximately 32% of spores failed to germinate (Figure 3A). Importantly, in this mutant strain, up to one-third of spores germinated, but hyphal tubes stopped extending within the first four h of their emergence, which was often followed by cell lysis (Figure S5 A, yellow arrow). This phenomenon was observed in ∼2% of spores in the wild-type and Δ*hupA* strains and 5% of spores in the TopA-depleted strain (Figure 3A). However, in 24-hour cultures of the TopA-depleted strains, growth defects became apparent, often leading to hyphal growth arrest and/or lysis (Figure S5B).

To test whether severe growth impairment eventually leading to cell lysis may be related to changes in chromosome organization in the hyphae, we examined chromosome distribution in the hyphae of the TopA-depleted strain and the TopA-depleted Δ*hupA* strain. DAPI staining of DNA showed that in the TopA-depleted Δ*hupA* mutant strain, the distance between the tip of the hyphae and the chromosome was much longer than in the wild-type or *hupA* deletion strains as well as the TopA-depleted control strain (Figure S5C, E). This finding could indicate that the elimination of *hupA* under elevated supercoiling conditions had a larger effect on chromosome distribution within the hyphae than the elimination of *hupA* under normal chromosome supercoiling.

Therefore, to further assess changes in chromosome distribution in *S. coelicolor* hyphae at the single-cell level, we compared the *oriC* distribution in the germinating hyphae of the TopA-depleted *hupA* mutant (TopA* *ΔhupA parB-egfp dnaN-mCherry*, ASMK05), the *hupA* mutant (*ΔhupA parB-egfp dnaN-mCherry*, ASMK02), the TopA-depleted strain (TopA* *parB-egfp dnaN-mCherry*, AS11) and the wild-type control strain (*parB-egfp dnaN-mCherry*, AK101) (Figure 3B). Our previous work showed that during the extension of *S. coelicolor* hyphae, the ParB-bound *oriC* region of the apical chromosome remained at a constant distance from the hyphal tip and that TopA depletion affected ParB positioning in vegetative hyphae (13,19). Here, all analysed strains contained ParB-EGFP, which binds to *parS* sites and serves as a marker for *oriC* localization, and DnaN-mCherry, which serves as a marker for replisome positions in hyphae.

The *oriC* positioning was highly disturbed in extending hyphae in the TopA-depleted strain and the TopA-depleted Δ*hupA* strain but not in the Δ*hupA* strain. The ParB complexes visible in the hyphae of both TopA-depleted strains (wild-type background and Δ*hupA*) appeared clustered together, further from the hyphal tip, than in the Δ*hupA* and control strains, where the ParB complexes were distributed along the hyphae (Figure 3B). In both TopA-depleted strains, the distance between the individual ParB complexes was significantly decreased (median distance 1.23 μm in the TopA-depleted strain and 1.36 μm in the TopA-depleted Δ*hupA* strain, compared to 1.55 μm and 1.62 μm for the control and Δ*hupA* strains) (Figure 3C).

The observed clustering of the ParB complexes suggested their inefficient separation after chromosome replication. The plot of the distances between the apical ParB complexes after the initial ParB complex duplication showed similarly ineffective chromosome separation in both TopA-depleted strains (wild-type background and Δ*hupA*), while in the control strain and Δ*hupA* strain, this distance increased during the first 60 min after duplication, reaching approximately 1.75 μm (Figure 3D). Moreover, the number of visible ParB complexes in the TopA-depleted strains appeared to be more variable than in either the reference or the Δ*hupA* strains (Figure S6). Unfortunately, DnaN-mCherry could not be analysed in germinating spores due to the weak fluorescence signal of the mCherry protein. The analysis of the ParB duplication combined with the DnaN-mCherry appearance (as an additional marker for chromosome replication) in the 24-h vegetative mycelium of the control and TopA-depleted strains showed that while the distance between the duplicated ParB complexes increased over time in the wild-type strain, this distance remained constant in the TopA-depleted strain, confirming ineffective ParB separation (Figure S7). To exclude the possibility that TopA depletion severely impaired DNA replication, we additionally analysed the DnaN-EGFP distribution in control and TopA-depleted strains (*dnaN-EGFP*, J3337 and TopA* *dnaN-EGFP*, AS07) (Figure S8). We observed only a slightly decreased number of DnaN-EGFP complexes accompanied by their higher fluorescence intensity during germination in the TopA-depleted strain compared to the control strain, indicating a lack of a significant impact of TopA depletion on replication.

The lack of *oriC* separation was also associated with the loss of apical nucleoid anchorage in TopA-depleted strains. After germination, the distance between the apical chromosome *oriC* and the hyphal tip remained constant in the control and Δ*hupA* strains but increased by ∼0.1 μm every 10 min in both TopA-depleted strains (wild-type background and *hupA* deletion) (Figure 3E). In longer hyphae (more than 10 μm), in both TopA-depleted strains, the distance between the tip and the apical o*riC* increased significantly, reaching median values of 4.17 and 4.86 μm, respectively, whereas this distance was 1.85 μm in both the control strain and the Δ*hupA* strain (Figure 3F).

In conclusion, a lower level of TopA in the vegetative mycelium of *S. coelicolor* inhibits the separation of the *oriC* regions after replication, leading to *oriC* clustering in the hyphae and the loss of chromosome anchorage at the hyphal tips. However, chromosome distribution in the TopA-depleted Δ*hupA* strain was only marginally more disturbed than in the TopA-depleted strain and did not fully explain the severely inhibited growth of the TopA-depleted Δ*hupA* strain.

### Impaired chromosome distribution in TopA-depleted strains inhibits hyphal extension

**Figure 4.**
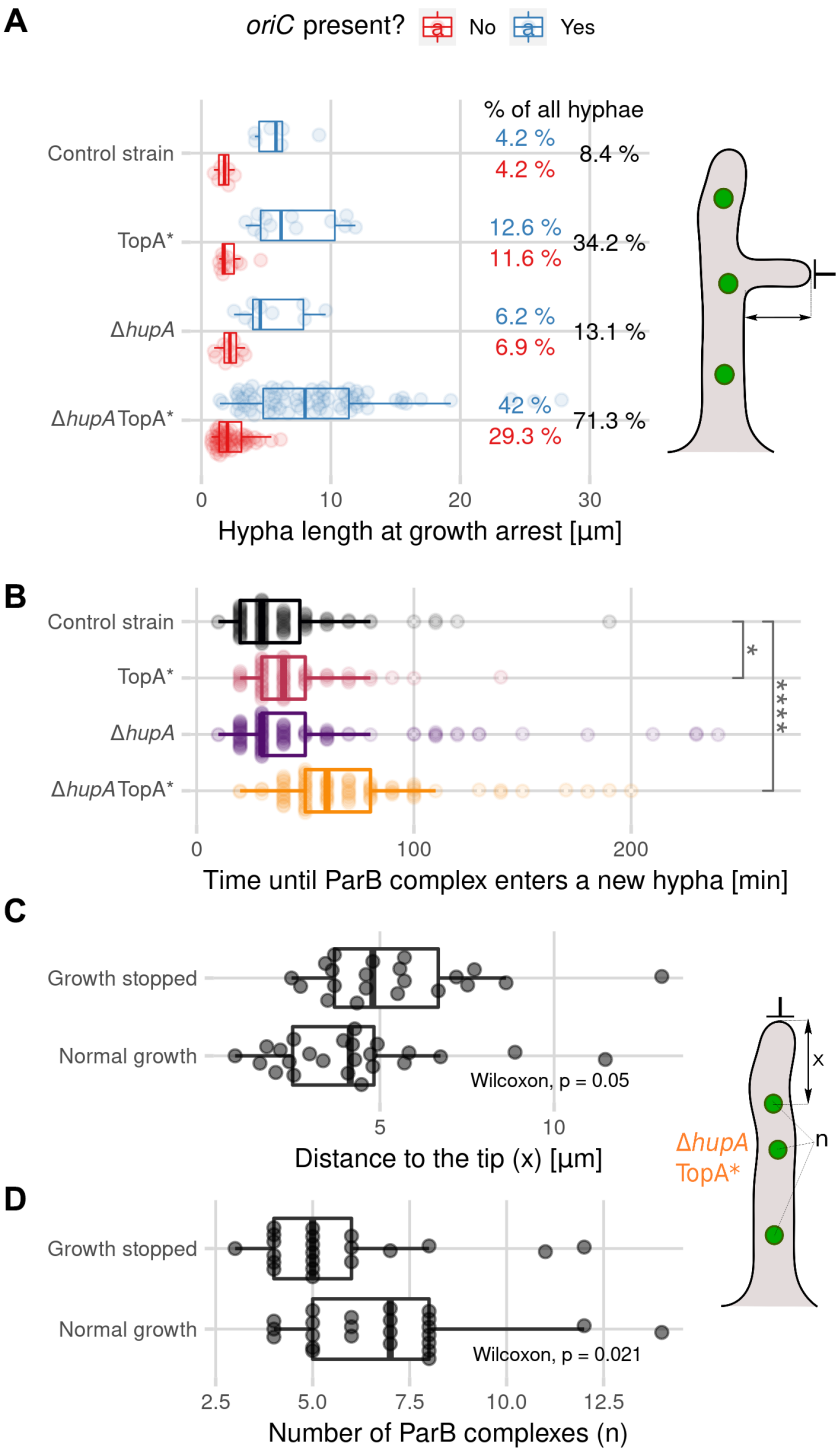
Correlation between disturbed chromosome targeting to the new hyphal branches and hyphal growth arrest. (**A**) Distribution of the stalled branch lengths that contained (blue) or lacked (red) the ParB complex (*oriC*) in the control strain (AK101, 144 hyphae), TopA-depleted strain (TopA*, AS11, 95 hyphae), Δ*hupA* strain (ASMK02, 145 hyphae) and TopA-depleted Δ*hupA* strain (TopA* Δ*hupA*, ASMK05, 174 hyphae) (all in the *parB-egfp* and *dnaN-mCherry* background), with the total percentage of stalled branches indicated in the plot (black). Hyphae were classified as stalled if no growth re-initiation was observed until the end of the experiment (16 h). The values are shown as semitransparent points, with boxplots showing the median values for the first and third quartiles for the strains. A schematic drawing of *S. coelicolor* hyphae shows the measured distance. (**B**) Distribution of the time intervals between the appearance of a new branch and the appearance of the first ParB complex within the new branch in the control strain (AK101, black, 144 hyphae), TopA-depleted strain (TopA*, AS11, pink, 95 hyphae), Δ*hupA* strain (ASMK02, purple, 145 hyphae) and TopA-depleted Δ*hupA* strain (TopA* Δ*hupA*, ASMK05, orange, 174 hyphae) (all in the *parB-egfp* and *dnaN-mCherry* genetic background). The values are shown as semitransparent points, with boxplots showing the median values for the first and third quartiles. (**C**) Distribution of the distances between the tip-proximal ParB complex and the hyphal tip (x) in hyphae in the TopA-depleted Δ*hupA* strain (TopA* Δ*hupA*, ASMK05), in which the growth was either stalled (21 hyphae) or continued normally (24 hyphae). (**D**) Distribution of the number of visible ParB complexes (n) in the hyphae of the TopA-depleted Δ*hupA* strain (TopA* Δ*hupA*, ASMK05), divided into two groups: stalled hyphae (21 hyphae) and normally extending hyphae (24 hyphae). Measurement of stalled hyphae for Panels C and D was performed 10 min before growth arrest and in normally growing hyphae of similar lengths. The boxplots show the median values for the first and third quartiles. A schematic drawing of the *S. coelicolor* hyphae shows the measured values for Panels C and D. In Panels B, C and D, the statistical significance was assessed using the Wilcoxon test, with the following significance levels: *P ≤ 0.05, **P ≤ 0.01, ***P ≤ 0.001, ****P ≤ 0.0001.

Our earlier studies showed that the loss of apical anchorage (due to *parA* or *parB* deletion) was correlated with inhibited chromosome migration from the hyphal stem to new branches and impaired branch growth (13). Thus, we expected that the impaired *oriC* distribution due to TopA depletion would also influence branch extension. In the TopA-depleted strain, the percentage of new branches that stopped growing (no extension for 120 min) increased to 24% from 8% in the wild-type strain. *hupA* deletion also inhibited the extension of 13% of new hyphae. TopA depletion combined with *hupA* deletion almost entirely abolished the formation of new branches, with more than 70% of branches exhibiting growth arrest (Figure 4A). This event was often accompanied by lysis and/or growth arrest of the maternal hyphae and other neighbouring branches (Figure S5 A, red arrows). Moreover, the fraction of hyphae lacking *oriC* increased from 4.2% in the wild-type strain to 29.3% in the TopA-depleted Δ*hupA* strain. In all studied strains, the hyphae that stopped growing and lacked the *oriC* signal (ParB complex) were significantly shorter than hyphae that contained at least one ParB complex (Figure 4A). In the reference strain and the Δ*hupA* strain, the first ParB complex was observed to migrate to the new branch shortly after it was established (median time 30 min). In the TopA-depleted strain and the TopA-depleted Δ*hupA* mutant strain, this value increased to 40 and 60 min, respectively (Figure 4B). This observation reinforces the idea that inefficient separation of multiple chromosomes impairs their migration to new hyphal branches and shows that the TopA-depleted Δ*hupA* mutant is the most severely affected in terms of chromosome targeting to the new hyphal branch.

To confirm that the inhibition of hyphal extension in the TopA-depleted strains was associated with impaired chromosome distribution in germinating spores and emerging branches, we divided the hyphae from the most affected TopA-depleted Δ*hupA* strain into two groups based on whether they were able to continue growing throughout the whole experiment. Next, we compared the distance between the *oriC* region and the hyphal tip in both groups of hyphae: those that continued extending and those that stopped extending. The mean length of the hyphae was similar in both groups (17.9 μm for growing hyphae and 18.9 μm for stalled hyphae). In hyphae with arrested growth, the distance between the ParB complex and the hyphal tip was significantly higher than in hyphae with normal growth, and the overall number of visible ParB foci was significantly lower in hyphae with arrested growth than in hyphae with normal growth (4.79 μm and 5 visible ParB complexes versus 4.14 μm and 7 visible ParB complexes) (Figure 4C, D). These results clearly indicated that growth arrest after *oriC* migration to the new hyphae or germination could be attributed to a greater distance between the hyphal tip and the nucleoid.

In summary, aberrant chromosome positioning in the hyphae can be linked to the growth arrest observed in the TopA-depleted strain and the TopA-depleted Δ*hupA* strain. Our experiments showed that while TopA depletion had a greater impact on the distribution of ParB complexes in the hyphae, in the TopA-depleted Δ*hupA* mutant strain, chromosome targeting to new branches was the most impeded action. The severely impaired growth of the new hyphal branches can account for the inhibited growth of the TopA-depleted Δ*hupA* strain.

## DISCUSSION

HupA, one of the two *Streptomyces* HU homologues, has been previously shown to be the most abundant NAP during *Streptomyces* vegetative growth (20–22). Here, we demonstrated increased affinity of HupA for supercoiled DNA. The HupA sequence is homologous to HU proteins from *E. coli* (38% and 40% amino acid sequence identity to HUα and HUβ, respectively) and other bacteria, which suggests that its function and biochemical properties should be similar to those proteins. The 4-fold higher (measured *in vitro*) affinity of *S. coelicolor* HupA to supercoiled DNA compared to relaxed DNA is similar to that shown for the *H. pylori* HU homologue (40). A high affinity constant was also reported for *E. coli* HU and equalled 2.5 μM when binding linear DNA molecules (4). Low affinity of HU to DNA means that a high HU concentration in the cell is required for sufficient chromosome coverage, and indeed, reported levels of HU homologues in several bacterial cell species are high (20 000–50 000 molecules/cell in *E. coli*, which constitutes a concentration of 30–80 μM, and 30 000–60 000 molecules/cell in *M. smegmatis*) (3,38). Moreover, *S. coelicolor* HupA could either introduce negative supercoils into relaxed DNA molecules in the presence of TopA or inhibit its activity (at 150 mM NaCl but not at 300 mM NaCl), similar to the *E. coli* HU heterodimer (36). Such features are common among HU homologues in prokaryotes (6,8,9,39–42). The mechanism proposed for *E. coli* involves the formation of HU octamers, around which 64–68 bp DNA fragments are wrapped (39). Recent studies have shown that in *E. coli*, 20 HU heterodimers are required to constrain a single negative supercoil (8) and that HU is responsible for higher negative supercoiling during *E. coli* stationary growth (43).

We also confirmed that HupA had an increased affinity for supercoiled DNA *in vivo*. In the ChIP-seq experiment, we found, depending on the analysis method used, 109 to 649 sites with enriched HupA binding only under high negative supercoiling conditions, and using PALM experiments, we found a decrease in HupA molecule mobility in the TopA-depleted strain, indicating an increase in the fraction of DNA-bound HupA. This trend was reversed when the TopA level was increased. The observed HupA binding corroborates earlier findings that HU homologues bind to DNA briefly (as shown by PALM experiments in *Bacillus subtilis* and *in vitro* experiments on *E. coli* HU), without sequence specificity and with a low affinity constant (with only a preference for A/T rich regions) (37,44–46). On the other hand, the diffusion coefficient obtained for the immobile HupA fraction was much lower than that calculated for *E. coli* HUα (0.015 μm^2^ s^-1^, 74.9% of molecules and 0.14 μm^2^ s^−^^1^, 43% of molecules, respectively), while the mobile fraction diffusion constants were more similar (0.4 μm^2^ s^−1^ and 0.39 μm^2^ s^−1^, respectively), possibly indicating a difference in how *S. coelicolor* HupA and *E. coli* HUα bind to DNA; however, this discrepancy may also be explained by differences in experimental conditions (47). Interestingly, while over 1000 HU binding sites were found on the *E. coli* chromosome (44), our analysis identified fewer HupA binding sites (∼120–427 under normal supercoiling, ∼230–1011 in the TopA-depleted strain, depending on the analysis method). This difference could be explained either by a high GC content of the *S. coelicolor* genome, by multiple chromosomal copies in the hyphae or by HupA-FLAG and native HupA competing for the same binding sites in our experiment. While the binding signal of HupA, similar to that of *E. coli* HU (44), was weak and difficult to differentiate from the background, HupA binding sites were characterized by higher AT% than neighbouring regions. Moreover, ChIP-seq analysis indicated that HupA preferentially bound to the central region of the chromosome. Our data show that HupA binding to the central chromosomal region did not depend on TopA levels and that sites bound only in TopA-depleted strains were often located further away from the *oriC* region. Increased HupA binding to the central part of the chromosome may be explained by the higher transcriptional activity of the genes located there (48,49); transcription generates negative supercoils, which are preferentially bound by HupA. This binding pattern was similar to that observed for HupB from *M. smegmatis* (38) but different from that observed for HupS binding to the *S. venezuelae* chromosome, which is enriched at chromosomal arms (15). This difference may indicate that the two HU homologues in *Streptomyces* bind to different chromosomal regions.

The absence of HupA was shown to cause only moderate growth retardation, similar to previous reports of *hupA* deletion in the closely related *S. lividans* (23). Growth retardation could be explained by the lower percentage of germinating spores observed in microscopy experiments, which could either result from a reduced rate of replication or altered gene transcription in the Δ*hupA* strain. Indeed, the influence of HupA on both processes was observed in *E. coli* and *Salmonella enterica* (44,50,51). The HUαα dimer is necessary for the proper maintenance of chromosomal architecture during growth and is essential for long-range DNA binding in *E. coli* (52,53). Possibly under normal supercoiling conditions, the lack of HupA may be compensated for by the presence of other NAPs in *S. coelicolor*, such as HupS (20–22).

Markedly, we found that combining TopA depletion with *hupA* deletion had an extremely detrimental effect. A similar effect was previously observed in the *E. coli* mutant (*topA10*), where disrupting the *hupA* gene was much less efficient in the *topA* mutant (*topA10*) than in the wild-type strain and deleting both *hupAB* genes was unsuccessful (12). While the ability of TopA to unwind overtly supercoiled DNA is crucial for cell survival (16), we hypothesized that the presence of HupA may prevent detrimental changes in the nucleoid structure when TopA is depleted. Previous studies have shown that TopA depletion induced a DNA damage response (20); however, due to the poor growth of the TopA-depleted Δ*hupA* strain, we could not estimate transcriptional changes resulting from such an altered nucleoid structure. The Δ*hupA* strain was also more sensitive to novobiocin treatment than the wild-type strain, indicating that HupA plays a role in the maintenance of topological homeostasis during excessive chromosome relaxation. The substantial fraction of unbound HupA (as shown by PALM) in *S. coelicolor* cells could presumably bind to DNA and stabilize plectonemic loops during supercoiling imbalance. We expected that increased HupA binding under high supercoiling would be accompanied by an increase in HupA levels, as suggested by previous transcriptional analysis (20). Surprisingly, our studies presented here did not confirm the increase in HupA protein levels after TopA depletion and even showed decreased HupA-FLAG and HupA-EGFP levels upon TopA depletion. We speculate that the reason for this phenomenon may be that fusion of the *hupA* gene modifies the regulation of *hupA* gene expression. In fact, we observed that the level of transcripts from tagged *hupA* genes was lower than that of the native gene, while *hupA* in the nonnative chromosomal locus in the complementation strain was expressed at the same level as the native *hupA* gene. These observations support the hypothesis of modified *hupA* gene expression, which could result from either altered transcriptional regulation that may involve autoregulation and supercoiling sensitivity of the *hupA* gene or modified transcript stability/transcriptional regulation of the fusion gene transcript. It should be noted, however, that in the wild-type strain, the impact of enhanced HupA binding combined with possibly increased HupA levels under increased chromosome supercoiling may be even more significant than observed in our studies.

We hypothesized that the impaired growth of the TopA-depleted Δ*hupA* strain was due to the deeply altered nucleoid structure and/or the impaired distribution of multiple *Streptomyces* nucleoids in the hyphae. Surprisingly, TopA depletion in the Δ*hupA* strain did not impair germination itself; in fact, the fraction of germinating spores was higher than when TopA was depleted in the wild-type background. Perhaps the lack of HupA combined with higher negative DNA supercoiling triggers germination, but the hyphal tubes still fail to extend due to problems with chromosome separation. The impaired separation of ParB complexes was previously observed in the TopA-depleted strain during sporulation (16). In both TopA-depleted and Δ*hupA* TopA-depleted strains, the position of the apical chromosome was considerably disturbed, but the distribution of chromosomal *oriC* regions located further from the hyphal tip was also affected, and *oriC* clustering was clearly detectable. These observations are consistent with our earlier report that the distance between the apical chromosome and the hyphal tips was higher in the TopA-depleted strain (19). Interestingly, the difference in the apical chromosome position between the TopA-depleted strain and the TopA-depleted Δ*hupA* strain was only significant in longer hyphae (> 10 μm). The clustering of neighbouring chromosomes within the hyphae in the TopA-depleted strain and the TopA-depleted Δ*hupA* mutant strain suggests impaired chromosomal separation after replication. This observation corresponds well with previous reports of the *E. coli* topoisomerase mutant phenotype, which is the formation of elongated cells with many unsegregated chromosomal copies (54). In *S. coelicolor*, the ParE/ParC system is primarily responsible for DNA decatenation, but it is not essential for replication of the linear chromosome. It cannot be excluded that TopA could also participate in this process (55).

The most critical step in vegetative mycelium growth is the formation of new branches, which requires the capture of the *oriC*-ParB complex by ParA at the tip of the newly established branch. The lack of ParA anchorage inhibits branch growth and chromosomal targeting to the new branch (13). Our data show that while TopA depletion and HupA deficiency both disturbed the growth of new branches, it was only when they were combined that new branch growth was severely slowed. The increased time between the appearance of new hyphae and the targeting of the chromosomes to the new branch indicates a problem with the chromosomal separation required for the successful capture of ParB by the ParA protein. Higher levels of DNA supercoiling may disrupt ParB capture by ParA, which is needed for proper chromosome anchorage to the growing hyphal tip. We suggest that the disrupted nucleoid separation results in unsuccessful branch formation and the frequent cell lysis observed during germination and early growth of the TopA-depleted Δ*hupA* strain. While transcriptional changes such as those observed in the TopA-depleted strain (19) undoubtedly contribute to the phenotype of the TopA-depleted Δ*hupA* strain, we infer that the disruption of nucleoid distribution accounts at least partially for this mutant strain growth inhibition.

In summary, we demonstrated that in *S. coelicolor*, DNA binding of the most abundant NAP, HupA, is elevated under increased chromosome supercoiling. The elimination of HupA significantly enhances the detrimental effects of the increased negative supercoiling due to TopA depletion, leading to inefficient nucleoid separation and, consequently, hyphal growth inhibition.

## DATA AVAILABILITY

The raw ChIP-Seq data generated in this study (shown in Figures 1 and S4) have been deposited in the ArrayExpress database (EMBL-EBI, https://www.ebi.ac.uk/biostudies/arrayexpress) under accession code E-MTAB-11369.

## Supplementary Material

gkac1093_Supplemental_File

## Notes

Present address: Agnieszka Kois-Ostrowska, Qiagen, Wroclaw, Poland.

Present address: Magda Kędra, International Institute of Molecular and Cell Biology, Warsaw, Poland.
