# Supplementary material for: Enhanced binding of an HU homologue under increased DNA supercoiling preserves chromosome organisation and sustains Streptomyces hyphal growth
Source: Nucleic Acids Res. 2022 Nov 24;50(21):12202–16. doi: 10.1093/nar/gkac1093 (PMC9756944; doi:10.1093/nar/gkac1093)
Supplement: gkac1093_Supplemental_File [file gkac1093_supplemental_file.pdf]

Supplementary Data

Table S1

Streptomyces coelicolor strains used in the study

Strain	Relevant genotype	Sources or reference
M145	SCP1- SCP2-	Bentley et al., 2002
PS04	M145 *ΔtopA::scar* attBΦC31::pIJ6902*topA*	Szafran et al., 2013
ASMK01	M145 *ΔhupA::hyg*	This study
ASMK03	PS04 *ΔhupA::hyg*	This study
ASMK011	M145 *ΔhupA::scar*	This study
ASMK031	PS04 *ΔhupA::scar*	This study
AK101	M145 *parB-egfp dnaN-mCherry::apra*	Kois-Ostrowska et al., 2016
AS11	AK101 *ΔtopA::hyg* attB φC31::pIJ6902*topA*	Strzałka et al., 2017
AS11.1	AK101 *ΔtopA::scar* attB φC31::pIJ6902*topA*	This study
ASMK02	M145 *parB-egfp dnaN-mCherry::apra ΔhupA::hyg*	This study
ASMK05	AK101 *ΔtopA::scar* attB φC31::pIJ6902*topA* *ΔhupA::hyg*	This study
ASMK012	M145 Δ*hupA::scar* pIJ170*hupA-FLAG*	This study
ASMK032	PS04 pIJ170*hupA-FLAG*	This study
ASMK034	PS04 *ΔhupA::scar* pIJ170*hupA-FLAG*	This study
ASMK015	M145 *ΔhupA::scar* pIJ170*hupAPAmCherry*	This study
ASMK035	PS04 *ΔhupA::scar* pIJ170*hupAPAmCherry*	This study
ASMK013	M145 *ΔhupA*::*scar* pIJ170*hupA*	This study
ASMK033	PS04 *ΔhupA*::*scar* pIJ170*hupA*	This study
ASMK016	M145 *ΔhupA*::*scar* pWHM3Hyg	This study
ASMK036	PS04 *ΔhupA::scar* pWHM3Hyg	This study
K306	M145 *hupA-egfp*	Salerno et al., 2009
AS40	M145 *hupA-egfp ΔtopA::scar* attBΦC31::pIJ6902*topA*	This study
MS10	M145 pWHM3Hyg	Szafran et al., 2016
MS11	PS04 pWHM3Hyg	Szafran et al., 2016
AS41	M145 pSS170*ermhupA*	This study
J3337	M145 *dnaN-EGFP*	Ruban-Ośmiałowska, 2006
AS07	PS04 *dnaN-EGFP*	This study

Table S2

Oligonucleotides used in the study

Primer name	Sequence
hupA_FW	GAACGATCCGCGCAGTCCCCGGCACTCCAAGCGGAGCGCTACGTAAT TCCGGGGATCCGTCGACC
hupA_RV	GGCACGCCTCAACGGCAAGAAGAACACGGGAGTAACAACTACGTAT GTAGGCTGGAGCTGCTTC
SLIC-hupA-FLAG_FW2	GACAAAACTTTAGCATGCAGGTACCCGAACTACATCATCCCCAG
SLIC-hupA-FLAG_RV2	AACCCTAGGGGATCCATCACTTGTCATCGTCATCCTTGTAATCGATGTC ATGAT
hupA_pam_long_FW	GAGGTTGAAAAACGCTCACTGGTACGAACTACATCATCCCCAG
hupA_pam_long_RV	TTGCTCACCATGTTAATTAAGGTACACTTGCCCTTGGCGGCTTC
pam_FW	TCACTGGTACCTTAATTAACATGGTGAGCAAGGGCGAG
pam_RV	CATCGATTCGCGACTTAAGCTTACTTGTACAGCTCGTC
SCO_HupA_promoter_Fw	AGCTCCATGGGGTACCCGAACTACATCATCCCCAGCG
SCO-HupA-FLAG_Rv	AGCTGGATCCCTTGCCCTTGGCGGCTTCC
HupASco_fw	GGATCCATGAACCGCAGTGAGCTGGTGGCC
HupASco_rv	CTCGAGCTTGCCCTTGGCGGCTTCCTTG
hupA_pss_FW	TCACTGGTACCTTAATTAACGAACTACATCATCCCCAG
hupA_pss_RV	CATCGATTCGCGACTTAAGCCTACTTGCCCTTGGCGGC
RT_sco2950_FW	CGGCGACATCGTCTCCAA
RT_sco2950_RV	TGCGCTCGAAAGTCAGGAA
hrdBRT_f	TGCTCTTCCTGGACCTCATC
hrdBRT_r	GTAGCCCTTGGTGTAGTCGAA
ermhupA_FW	AGGAGGCCCCATATGAGATCATGAACCGCAGTGAGCTGG
ermhupA_RV	GACTCTAGTTAATTAATCACCTACTTGCCCTTGGCGGC

Strains construction and protein purification

Construction of *hupA* deletion strains

To construct the *S. coelicolor* strain lacking the *hupA* gene, first we constructed a E59 cosmid derivative containing hygromycin resistance cassette instead of *hupA* gene using primers hupA_FW and hupA_RV and PCR targeting method, which yielded E59 *ΔhupA::hyg* cosmid. Secondly hygromycin resistance cassette was removed by SnaBI digestion and religation to create E59 *ΔhupA::scar* cosmid. In this cosmid ampicillin resistance cassette in SuperCos was replaced with hygromycin resistance cassette containing *oriT* site, necessary for conjugation into *S. coelicolor.*

In order to complement *hupA* deletion a 769 bp long fragment containing *hupA* gene and its promoter sequence was amplified using hupA_FW and hupA_RV primers and then cloned into pIJ170 at KpnI site with the SLIC method yielding pIJ170 *hupA* plasmid. After verification with sequencing this vector was introduced into ASMK011 (ΔhupA::scar) and ASMK031 (ΔhupA::scar, TopA depletion) strains in order to obtain ASMK013 and ASMK033 strains, respectively.

In order to remove hygromycin resistance cassette from AS11 (TopA*, *parb-egfp, dnaN-mcherry*) strain we used a H5 *ΔtopA::scar* cosmid. After conjugation colonies sensitive to hygromycin and kanamycin were obtained indicating a successful double crossing-over yielding strain AS11.1.

The cosmid E59*ΔhupA::hyg* was introduced into *S. coelicolor* strains M145, PS04 (TopA-controlled), AK101 (*parb-egfp, dnaN-mcherry*) and AS11.1 (TopA-controlled, *parb-egfp, dnaN-mcherry*). hygromycin resistant and kanamycin sensitive clones were selected indicating a successful double crossing-over. The obtained strains: ASMK01 (*ΔhupA::hyg*), ASMK03 (*ΔhupA::hyg*, TopA depletion), ASMK02 (*ΔhupA::hyg, parb-egfp, dnaN-mcherry*) and ASMK05 (*ΔhupA::hyg*, TopA-controlled, *parb-egfp, dnaN-mcherry*) were verified by PCR. In order to remove hygromycin resistance cassette from ASMK01 and ASMK03 strains we used a E59 *ΔhupA::scar* cosmid. After conjugation colonies sensitive to hygromycin and kanamycin were obtained indicating a successful double crossing over yielding strains ASMK011 (*ΔhupA::scar*) and ASMK031 (*ΔhupA::scar*, TopA*), which were verified using PCR.

HupA-FLAG construction

773 bp long fragment containing *hupA* gene with its promoter sequence was amplified with primers SCO_HupA_promoter_Fw and SCO-HupA-FLAG_Rv. The obtained sequence was cloned into the pGEM-*SMC-FLAG* plasmid at NcoI-BamHI sites using the SLIC method, replacing the SMC gene. Then fragment containing *hupA-FLAG* gene and promoter sequence was amplified using SLIC-hupA-FLAG_FW2 and SLIC-hupA-FLAG_RV2 primers and cloned into pIJ170 integrative plasmid at XmaJI site using the SLIC method yielding pIJ170 *hupA-FLAG* plasmid. This vector, after verification with sequencing, was used to transform strain ASMK011 (Δ*hupA*) , PS04 (TopA*) and ASMK031 (*ΔhupA* TopA*). Obtained colonies resistant for hygromycin were verified using Western blot for production of HupA-FLAG protein yielding the strains ASMK012, ASMK032 and ASMK034.

HupA-PAmCherry construction

First, 751 bp long fragment containing *PAmCherry* gene was amplified with pam_FW and pam_RV primers and cloned into pIJ170-FLAG at XhoI and XmaJI site to obtain pIJ170 *PAmCherry* vector. Next, a 769 bp long fragment containing *hupA* gene and its promoter sequence was amplified using hupA_pam_long_FW and hupA_pam_long_RV primers and then cloned into pIJ170 *PAmCherry* at KpnI site with the SLIC method yielding pIJ170 *hupAPAmCherry* plasmid. After verification with sequencing this vector was introduced into ASMK011 (*ΔhupA::scar*) and ASMK031 (*ΔhupA::scar*, TopA*) strains in order to obtain ASMK015 and ASMK035 strains respectively.

ermhupA construction

First, 322 bp long fragment containing *hupA* gene was amplified with ermhupA_FW and ermhupA_RV primers and cloned into pIJ10257 (contains constitutive promoter *erm*) vector digested with XhoI using the

SLIC method. After verification with sequencing pIJ10257 *ermhupA* vector was introduced into M145 strain yielding strain AS41.

TopA* dnaN-EGFP and TopA* HupA-EGFP strains construction

First, pIJ6902 *ptiptopA* plasmid was introduced to strains K306 (Salerno et al., 2009) and J3337 (Ruban-Ośmiałowska, 2006). Than *topA* gene was deleted using H5*topA::scar* vector yielding strains AS40 (*ptiptopA ΔtopA::scar hupA-EGFP*) and AS07 (*ptiptopA ΔtopA::scar dnaN-EGFP*).

$TopA_{sv}$ purification

E. coli strain containing pET28*topAsv* was used for protein production. For protein overproduction, cells were grown to OD600 ∼ 0.4 at 37°C, then isopropyl-β-d-thiogalactopyranoside (IPTG) was added to a final concentration of 0.3 mM and the culture was continued for 4 h at 37°C. The cells were collected by centrifugation, re-suspended in 50 mM NaH_2PO_4, pH 8.1, 300 mM NaCl with 20 mM imidazole and sonicated. Fast protein liquid chromatography (FPLC) system with HisTrap HP columns (GE Healthcare) was used to purify recombinant proteins from cell lysate, followed by desalting using Zeba Spin Desalting Column (Thermo Scientific) equilibrated with 50 mM NaH_2PO_4, pH 8.1, 300 mM NaCl, 10% glycerol buffer. Protein samples were stored in -80°C.

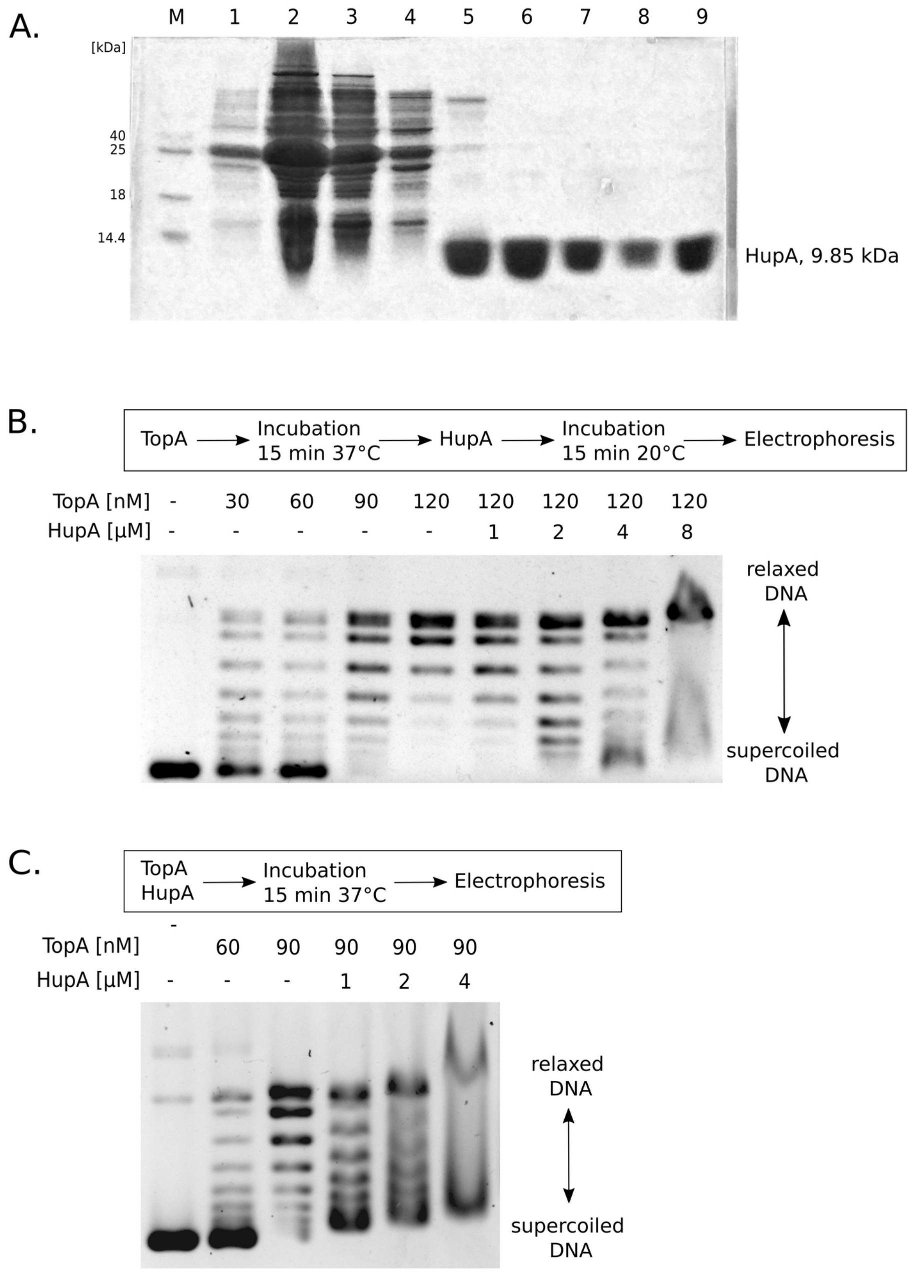

Fig. S1

A. Purification of HupA-GST protein. M – marker, 1 – culture lysate, 2 – cell pellet, 3-4 – column wash, 5-8 wash after PreScission protease treatment, 9 – HupA protein after desalting.

B. TopA relaxation assay followed by incubation with HupA. Two hundred nanograms of supercoiled plasmid pUC19 was initially incubated with TopA (30-120 nM) for 15 minutes, followed by the addition of HupA (0–8 µM) and incubation for 15 min at 20°C. Topoisomers were resolved without deproteinization by agarose gel electrophoresis.

C. TopA relaxation assay in the presence of HupA. Two hundred nanograms of supercoiled plasmid pUC19 was initially incubated with TopA (60-90 nM) and HupA (0–4 µM) for 15 minutes. Topoisomers were resolved without deproteinization by agarose gel electrophoresis.

The positions of the supercoiled and relaxed topoisomers are indicated.

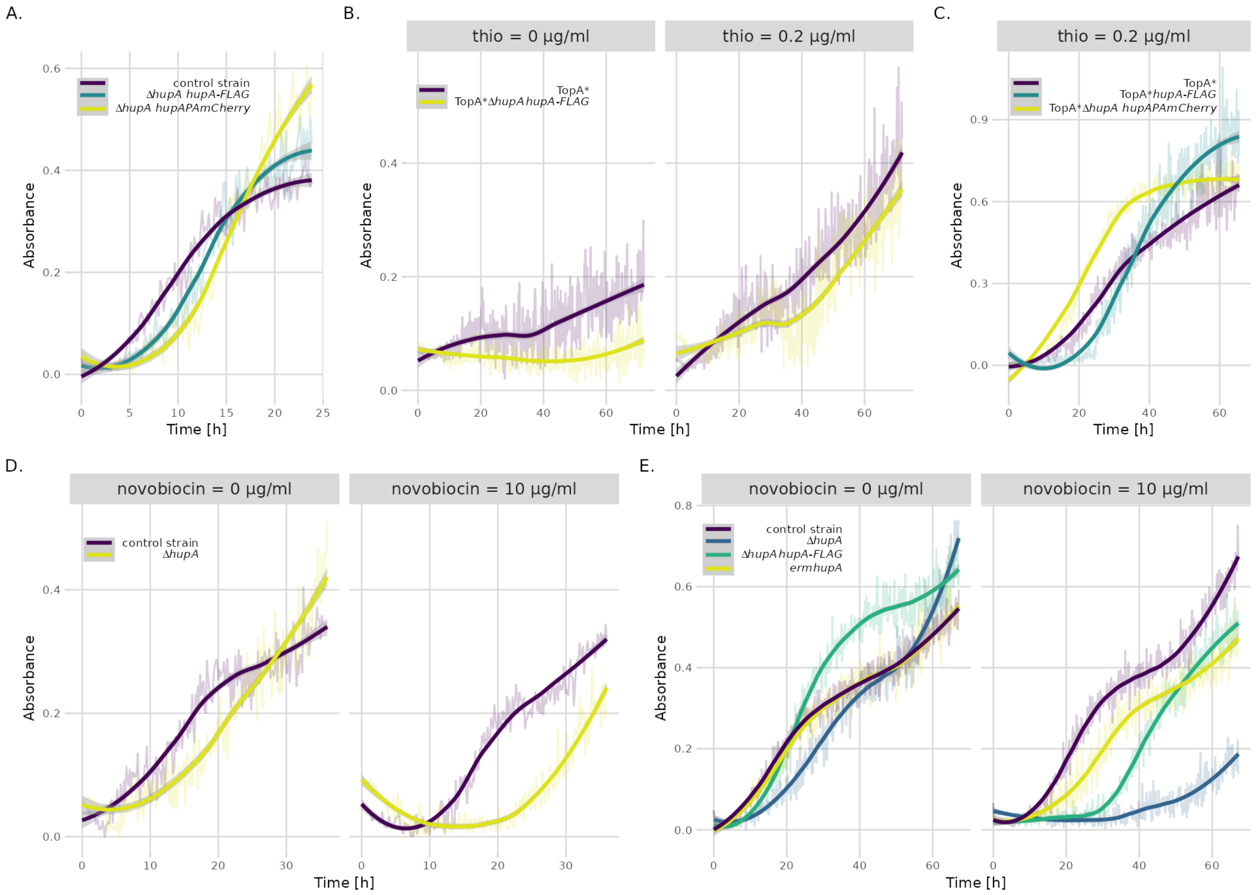

Fig. S2

A. Growth curves of the control strain (M145, purple), Δ*hupA hupA-FLAG* strain (ASMK012, green) and Δ*hupA hupA-PAmCherry* (ASMK015, yellow), cultured in ‘79’ medium.

B. Growth curves of the TopA-depleted strain (PS04, purple) and TopA-depleted Δ*hupA hupA-FLAG* (TopA* Δ*hupA hupA-FLAG*, ASMK034, yellow), cultured in ‘79’ medium with various concentrations of *topA* inducer (thiostrepton: 0 or 0.2 µg/ml).

C. Growth curves of the TopA-depleted strain (TopA*, PS04, purple), TopA-depleted *hupA-FLAG* strain (TopA* *hupA-FLAG*, ASMK032, green) and TopA-depleted Δ*hupA hupA-PAmCherry* strain (TopA* Δ*hupA hupA-PAmCherry*, ASMK035, yellow), cultured in ‘79’ medium in the presence of a *topA* inducer (thiostrepton 0.2 µg/ml).

D. Growth curves of the Δ*hupA* (ASMK011, yellow) and control (M145, purple) strains, cultured in ‘79’ medium in the presence of gyrase inhibitor (novobiocin: 0 or10 µg/ml).

E. Growth curves of the control strain (M145, purple), Δ*hupA* strain (ASMK011, blue), Δ*hupA hupA-FLAG* (ASMK012, green) and *ermhupA* strain (AS41, yellow), cultured in ‘79’ medium.

The semitransparent lines show the mean absorbance values obtained from five replicates, and the bold lines correspond to the fit of the loess model.

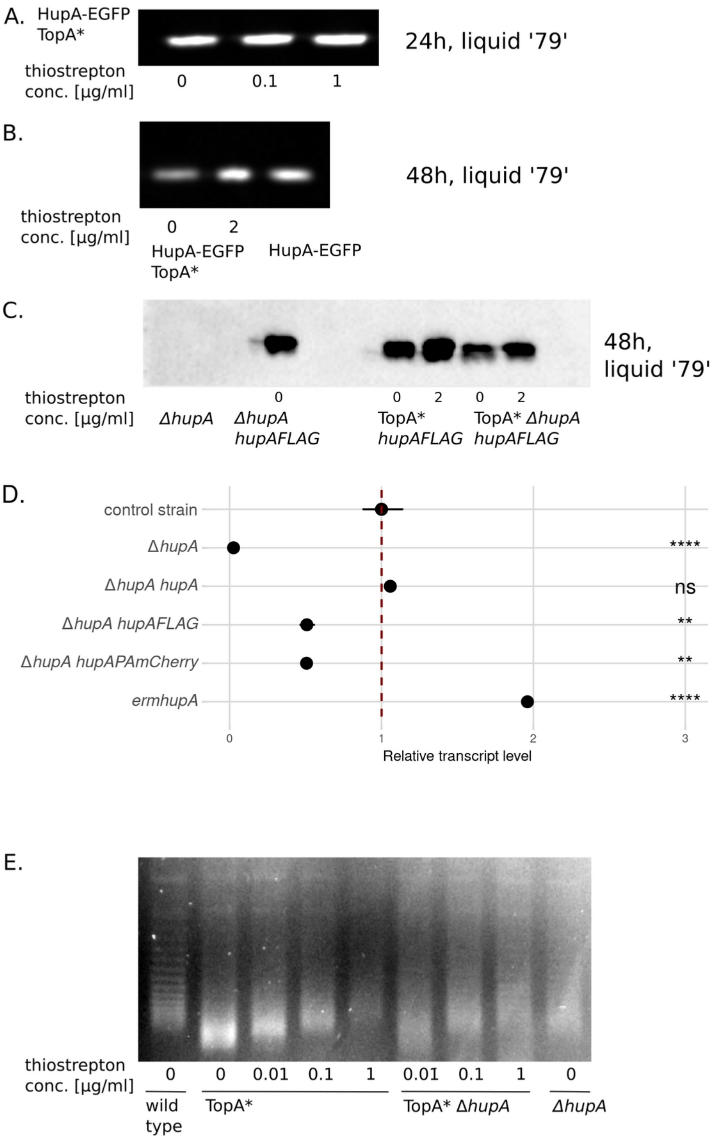

Fig. S3

A. HupA-EGFP fluorescence measured in native SDS PAGE gel using Azure 600 (Biosystems). Cell lysates from the TopA-depleted *hupA-EGFP* strain (TopA*, AS40*)* were prepared after 18 h of culture at 30°C. The protein concentration in each lane was normalized.

B. HupA-EGFP fluorescence measured in native SDS PAGE gel using Azure 600 (Biosystems). Cell lysates from the TopA-depleted *hupA-EGFP* strain (TopA*, AS40*)* compared to control strain *hupA-EGFP* (K306) were prepared after 48 h of culture at 30°C. The protein concentration in each lane was normalized.

C. Western blot results produced with an anti-FLAG antibody using cell lysates of strains Δ*hupA* (ASMK011), Δ*hupA hupA-FLAG* (ASMK012), TopA-depleted *hupA-FLAG* (TopA* *hupA-FLAG,* ASMK032) and TopA-depleted Δ*hupA hupA-FLAG* (TopA* Δ*hupA hupA-FLAG,* ASMK034) cultured for 48 h in '79' medium.

D. RT-PCR results of *hupA* gene expression in strains: wild type (M145), Δ*hupA* (ASMK011), Δ*hupA hupA* (ASMK013), Δ*hupA hupa-FLAG* (ASMK012), Δ*hupA hupA-PAmCherry* (ASMK015) and *ermhupA* (AS41). Strains were cultured in YEME/TSB medium for 24h. Each sample was performed in triplicate. Statistical analysis was performed using ANOVA with Tukey post-hoc test, statistical significance is given against the wild type strain.

E. Topoisomers distribution of plasmid pWHM3Hyg isolated from 48 h of culture of the control strain (MS10), TopA-depleted strain (TopA*, MS11), Δ*hupA* strain (ASMK016) and TopA-depleted Δ*hupA* strain (TopA* Δ*hupA,* ASMK036). The thiostrepton concentration (range 0-1 µg/ml) is indicated below the image.

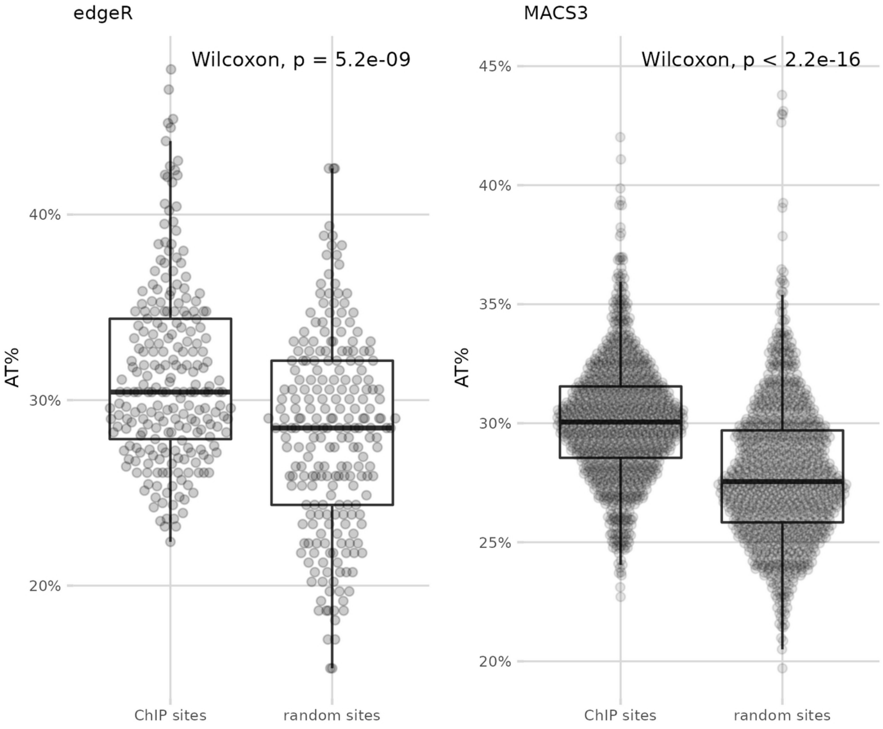

Fig S4

Comparison of AT% percent calculated for ChIP-seq HupA binding sites identified by edgeR (left) and MACS3 (right) and the same number of random *S. coelicolor* sequences of similar length, p-values calculated by two-sided Wilcoxon test is shown on the plot.

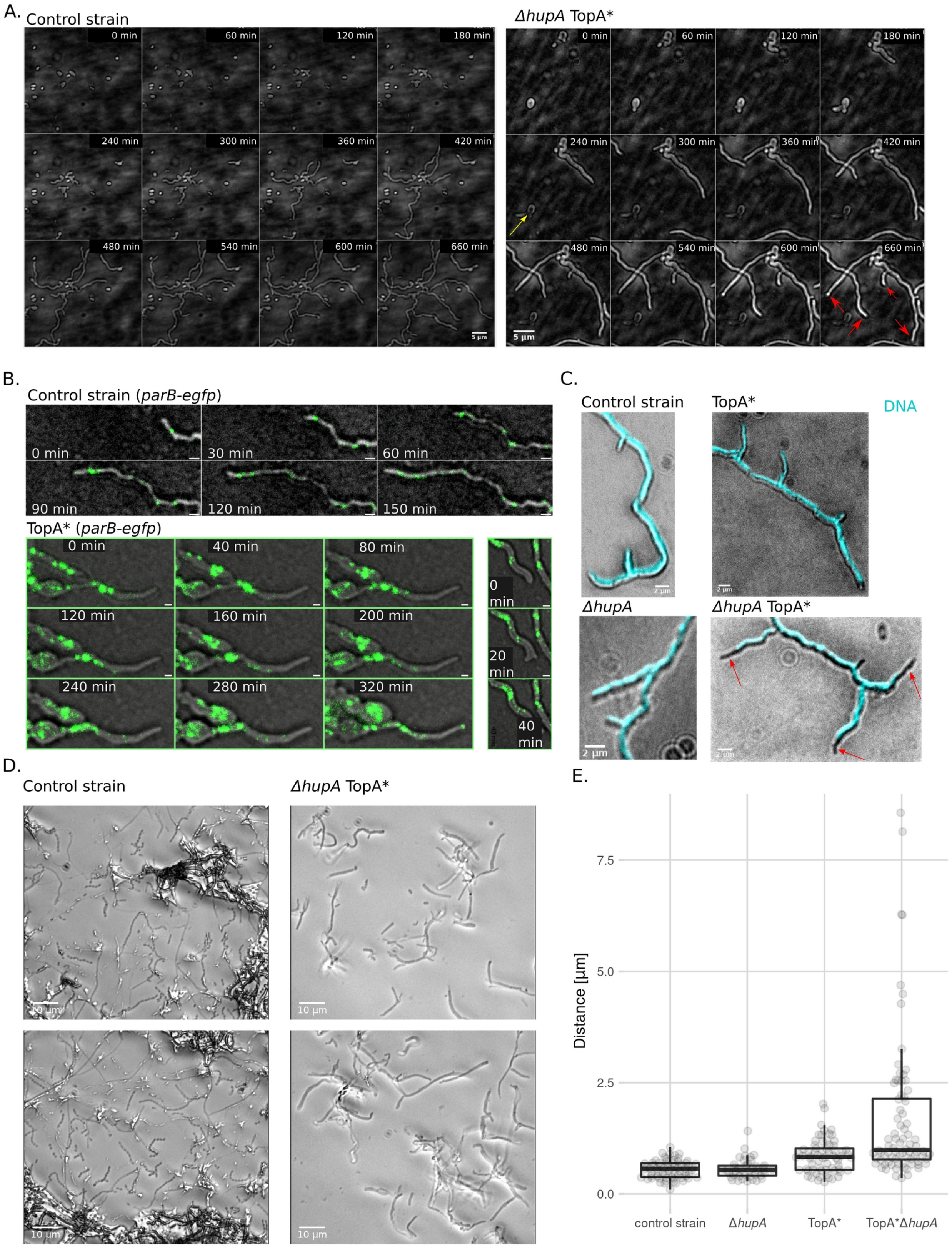

Fig. S5

A. Time-lapse DIC snapshots of germinating spores of the control strain (AK101, left panel) and TopA-depleted Δ*hupA* strain background (TopA* Δ*hupA*, ASMK05, right panel). The yellow arrow indicates a lysed geminating spore, while the red arrows indicate hyphae with arrested growth. Scale bar 5 µm.

B. Time-lapse DIC snapshots of 24 h hyphae of the control strain (AK101, top panel) and TopA-depleted strain (TopA*, AS11, bottom panels) overlaid with green fluorescence channels showing the ParB-EGFP complexes. Scale bar 1 μm.

C. Images of vegetative hyphae (DIC, grey) and DNA stained with DAPI (blue) of the Δ*hupA* (ASMK011), TopA-depleted (TopA*, PS04), TopA-depleted Δ*hupA* (TopA* Δ*hupA*, ASMK031, thiostrepton 0.01 μg/ml) and wild-type (M145) strains. Red arrows indicate hyphae lacking DNA in the tip proximal region. Scale bar 2 μm.

D. 7 days colony surface images of control strain (M145) and TopA-depleted Δ*hupA* strain (TopA* Δ*hupA*, ASMK031). Scale bars 10 μm.

E. Boxplots showing comparison of the distance between hyphal tip and the edge of DAPI signal in strains: control strain (M145, 65 hyphae), Δ*hupA* (ASMK011, 41 hyphae), TopA-depleted (TopA*, PS04, 66 hyphae) and TopA-depleted Δ*hupA* (TopA* Δ*hupA*, ASMK031, thiostrepton 0.01 μg/ml, 80 hyphae)

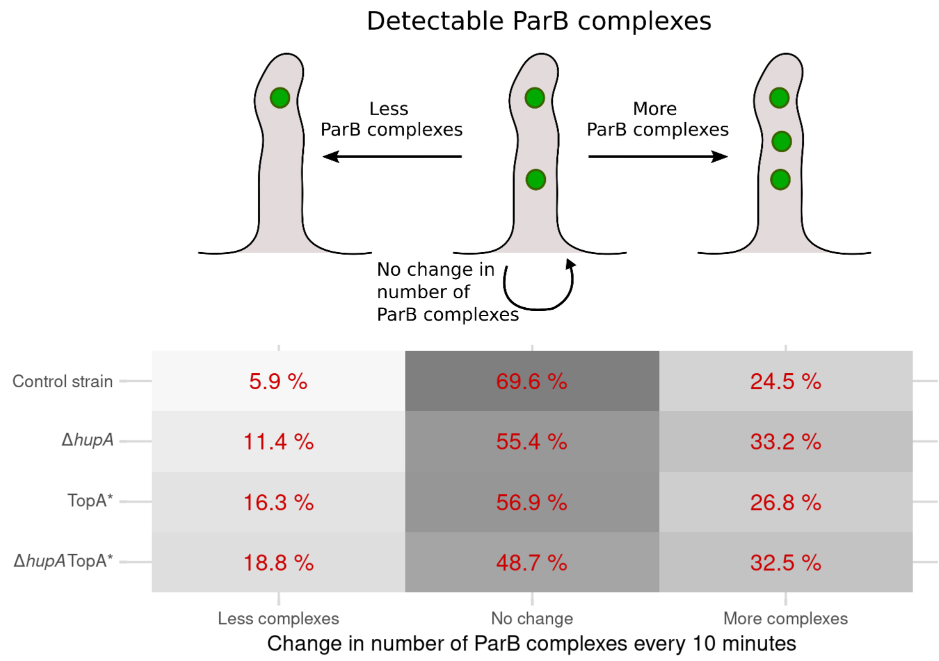

Fig. S6

Variability of the number of ParB complexes in germinating *S. coelicolor* hyphae in the control strain (AK101), TopA-depleted strain (TopA*, AS11), Δ*hupA* strain (ASMK02) and TopA-depleted Δ*hupA* strain (TopA* Δ*hupA*, ASMK05) (all in *parB-egfp* and *dnaN-mcherry* genetic background). The number of visible ParB complexes was compared between each consecutive time point (10 minutes), and the percentage of detectable ParB complex loss or gain was calculated.

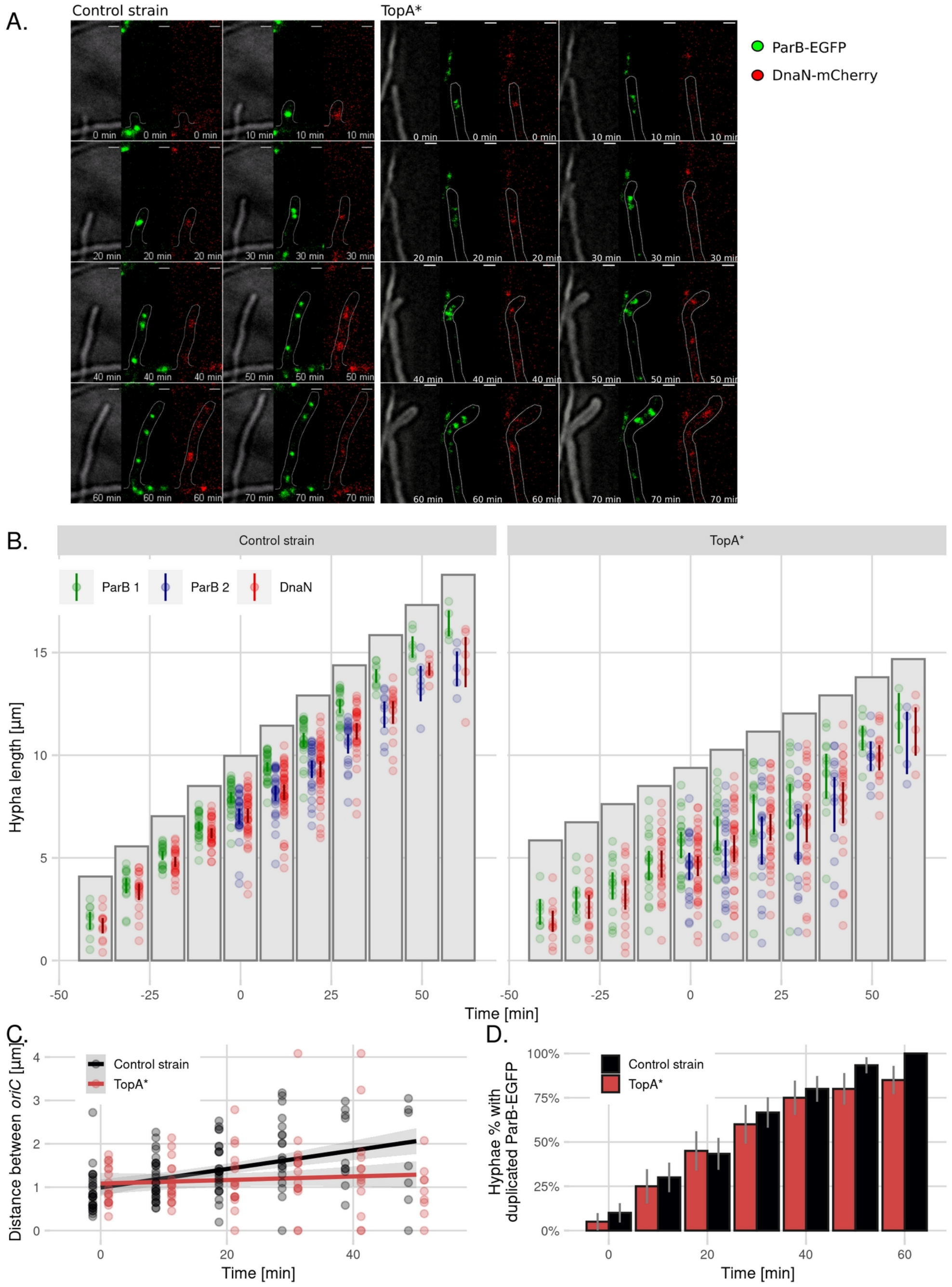

Fig. S7

A. Snapshots from the time-lapse analysis of the ParB-EGFP (green) and DnaN-mCherry complexes (red) in vegetative hyphae of the control strain (AK101) and TopA-depleted strain (TopA*, AS11). The fluorescence images are next to the DIC images (grey). Scale bar 1 µm.

B. Positions of the ParB-EGFP complexes adjacent to the tip (tip-proximal ParB1 – green and tip-distal ParB2 – blue) and DnaN-mCherry complexes (red) in hyphae (grey bar) of the control strain (AK101, 30 hyphae) and TopA-depleted strain (TopA*, AS11, 20 hyphae). For each timepoint, a mean with a 95% confidence interval is plotted.

C. Average distance between the ParB complexes after duplication over time in the control strain (AK101, black) and TopA-depleted strain (TopA*, AS11, red). Lines show the linear model with 95% confidence intervals.

D. Percentage of hyphae in which duplicated ParB complexes could be detected at the indicated time after replisome appearance in the control strain (AK101, black) and TopA-depleted strain (TopA*, AS11, red). Error bars show 95% confidence intervals.

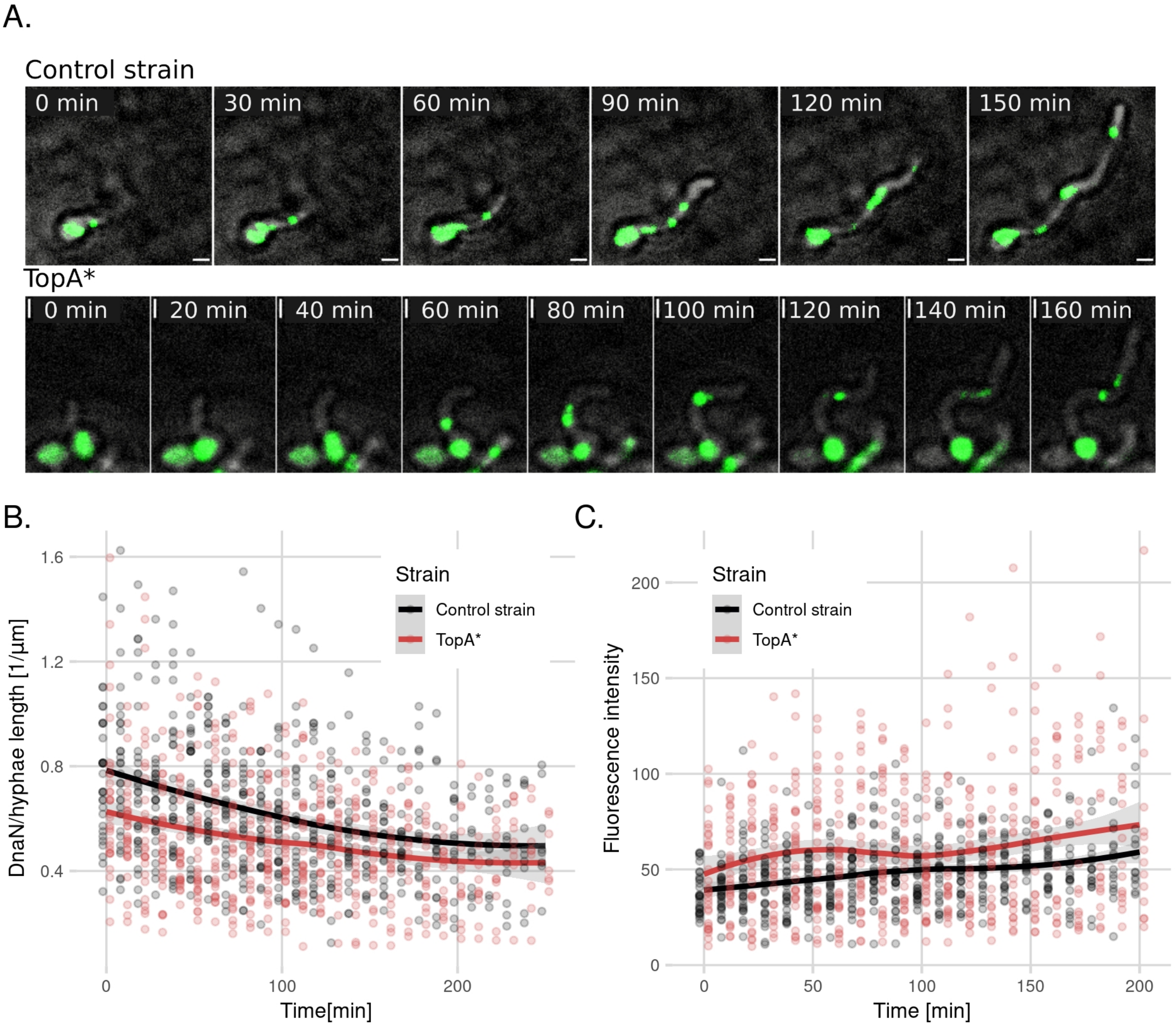

Fig. S8

A. Snapshots from the time-lapse analysis of the DnaN-EGFP (green) in germinating spores of the control strain (J3337) and TopA-depleted strain (TopA*, AS07). The fluorescence images are overlayed with the DIC images (grey). Scale bar 1 μm.

B. Number of DnaN-EGFP complexes divided by hyphae length over time for the control strain (J3337, black, 30 hyphae) and TopA-depleted strain (TopA*, AS07, red, 30 hyphae). Shown curve was fitted using loess algorithm.

C. Average fluorescence intensity of DnaN-EGFP complexes over time for the control strain (J3337, black, 30 hyphae) and TopA-depleted strain (TopA*, AS07, red, 30 hyphae). Shown curve was fitted using loess algorithm.
